# Chikungunya virus infection in Indonesia: a systematic review and evolutionary analysis

**DOI:** 10.1186/s12879-019-3857-y

**Published:** 2019-03-12

**Authors:** Harapan Harapan, Alice Michie, Mudatsir Mudatsir, Roy Nusa, Benediktus Yohan, Abram Luther Wagner, R. Tedjo Sasmono, Allison Imrie

**Affiliations:** 1 Medical Research Unit, School of Medicine, Universitas Syiah Kuala, Banda Aceh, Indonesia; 2 School of Biomedical Sciences, University of Western Australia, 35 Stirling Highway, Crawley, 6009 Australia; 3 Department of Microbiology, School of Medicine, Universitas Syiah Kuala, Jl. T. Tanoeh Abe, Darussalam, Banda Aceh, 23111 Indonesia; 4 Vector Borne Disease Control, Research and Development Council, Ministry of Health of the Republic of Indonesia, Jakarta, Indonesia; 5 Eijkman Institute for Molecular Biology, Jakarta, Indonesia; 6 Department of Epidemiology, University of Michigan, Ann Arbor, MI USA; 7 Pathwest Laboratory Medicine Western Australia, Nedlands, Western Australia Australia

**Keywords:** Chikungunya, Chikungunya virus, ECSA genotype, Indonesia, Systematic review

## Abstract

**Background:**

Despite the high number of chikungunya cases in Indonesia in recent years, comprehensive epidemiological data are lacking. The systematic review was undertaken to provide data on incidence, the seroprevalence of anti-Chikungunya virus (CHIKV) IgM and IgG antibodies, mortality, the genotypes of circulating CHIKV and travel-related cases of chikungunya in the country. In addition, a phylogenetic and evolutionary analysis of Indonesian CHIKV was conducted.

**Methods:**

A systematic review was conducted to identify eligible studies from EMBASE, MEDLINE, PubMed and Web of Science as of October 16th 2017. Studies describing the incidence, seroprevalence of IgM and IgG, mortality, genotypes and travel-associated chikungunya were systematically reviewed. The maximum likelihood phylogenetic and evolutionary rate was estimated using Randomized Axelerated Maximum Likelihood (RAxML), and the Bayesian Markov chain Monte Carlo (MCMC) method identified the Time to Most Recent Common Ancestors (TMRCA) of Indonesian CHIKV. The systematic review was registered in the PROSPERO database (CRD42017078205).

**Results:**

Chikungunya incidence ranged between 0.16-36.2 cases per 100,000 person-year. Overall, the median seroprevalence of anti-CHIKV IgM antibodies in both outbreak and non-outbreak scenarios was 13.3% (17.7 and 7.3% for outbreak and non-outbreak events, respectively). The median seroprevalence of IgG antibodies in both outbreak and non-outbreak settings was 18.5% (range 0.0–73.1%). There were 130 Indonesian CHIKV sequences available, of which 120 (92.3%) were of the Asian genotype and 10 (7.7%) belonged to the East/Central/South African (ECSA) genotype. The ECSA genotype was first isolated in Indonesia in 2008 and was continually sampled until 2011. All ECSA viruses sampled in Indonesia appear to be closely related to viruses that caused massive outbreaks in Southeast Asia countries during the same period. Massive nationwide chikungunya outbreaks in Indonesia were reported during 2009–2010 with a total of 137,655 cases. Our spatio-temporal, phylogenetic and evolutionary data suggest that these outbreaks were likely associated with the introduction of the ECSA genotype of CHIKV to Indonesia.

**Conclusions:**

Although no deaths have been recorded, the seroprevalence of anti-CHIKV IgM and IgG in the Indonesian population have been relatively high in recent years following re-emergence in early 2001. There is sufficient evidence to suggest that the introduction of ECSA into Indonesia was likely associated with massive chikungunya outbreaks during 2009–2010.

**Electronic supplementary material:**

The online version of this article (10.1186/s12879-019-3857-y) contains supplementary material, which is available to authorized users.

## Background

Arboviruses (*ar*thropod-*bo*rne viruses) are a group of viruses that exist in a transmission cycle between blood-feeding arthropod vectors and amplifying, vertebrate hosts. With most arboviruses, human involvement in this transmission cycle is incidental [1]. In terms of public health significance, the mosquito is the most important vector of arbovirus transmission. It is estimated that approximately 3.9 billion people, living in more than 120 different countries, are at risk of becoming infected with any of the three major arboviruses: Chikungunya virus (CHIKV), Dengue virus (DENV) and Zika virus (ZIKV) [2]. Chikungunya virus, which is primarily transmitted by *Aedes aegypti* and *Ae. albopictus* mosquitoes*,* is a positive-sense single-stranded RNA virus and a member of the family *Togaviridae* [3, 4]. The first well-characterised chikungunya outbreak was reported in Southern province, Tanganyika territory of Tanzania in 1952 [5, 6]. Sporadic chikungunya outbreaks were subsequently identified in parts of Africa and Asia during the 1950s and 1960s, followed by an apparent re-emergence in the 2000s [7]. Since 2005, large-scale outbreaks of chikungunya sweeping across south-western Indian Ocean and Southeast Asia [8–20]. In La Réunion, the outbreak affected about a third of the population [9, 21] and in India, the viruses infected more than 1.3 million persons during 2005–2006 [14]. In Sri Lanka, the viruses infected more than 100,000 people [15] and CHIKV subsequently spread to Southeast Asia including Indonesia.

The illnesses caused by CHIKV and DENV are clinically indistinguishable and the accurate diagnosis of these infections on clinical grounds alone is usually problematic [22, 23]. Although prior literature has stated that a higher proportion of people infected with CHIKV are symptomatic than those infected with DENV [24], a recent systematic review revealed that asymptomatic chikungunya had a very high variability in percentages ranging from 3.2% in La Réunion (2005–2006) to 82.1% in the Philippines (2012–2013) [25]. The highest percentage of asymptomatic chikungunya have been recorded with the Asian genotype in the Philippines with 82.1% [26]. The common symptoms of chikungunya include rash, high fever, severe joint and muscle pain, headache and photophobia [7, 27]. Severe symptoms, involving vital organs, may develop during CHIKV infection such as encephalitis [28, 29], encephalopathy [29–31], optic neuropathy [29, 32], neuroretinitis [32], myelopathy and myelitis [29], Guillain-Barré syndrome [29, 32], myocarditis [31], hepatitis [33], acute interstitial nephritis [34], severe sepsis [35], septic shock [35] and multi-organ failure [31–33, 36, 37]. In rare cases, infections may be fatal [28, 33–35, 37]. Perinatal CHIKV infection can cause sequelae such as microcephaly and cerebral palsy [38]. In adults, persisting arthralgia/arthritis, alopecia and depression are the most commonly recorded sequelae [39–42]. A meta-analysis found that approximately 25% of chikungunya cases develop chronic inflammatory rheumatism and 14% develop chronic arthritis [43], creating a major burden on society in terms of morbidity and economic productivity [41, 42, 44, 45].

CHIKV has an approximately 12 kb genome that encodes four non-structural proteins (NSP1–4) and five structural proteins (C, E3, E2, 6 K, and E1) [4]. Genetic analysis has identified three genotypes of CHIKV: the West African, East/Central/South African (ECSA), and Asian genotype [46]. The ECSA genotype consists of three lineages: Central African, East/South African, and the Indian Ocean lineage. These genotypes are spreading sporadically worldwide, with ECSA and Asian genotypes being the predominately isolated [47].

In Indonesia, based on official documents from the Ministry of Health (MoH), chikungunya cases were reported for the first time in Samarinda (Kalimantan island) in 1973 [48]. However, there is evidence to suggest that CHIKV infections have occurred in Indonesia prior to 1973. Serum samples collected between 1969 and 1972 demonstrated significant titres of anti-CHIKV antibodies when tested using haemagglutination inhibition assay (HI) and the plaque reduction neutralization test (PRNT) in most of the Indonesian archipelago, except Java [49, 50]. In addition, evidence from historical reports suggest that the first circulation of CHIKV was back in 1779 in Jakarta, when this infection called as *kidinga pepo* [51, 52]. This is widely acknowledged by experts in the field of arboviruses as the first report of chikungunya in Indonesia, although it is impossible to demonstrate by molecular clock analysis [53]. The first virologically confirmed chikungunya outbreak was reported in June 1982 in Jambi province of Sumatra island, followed by multiple outbreaks between 1983 and 1984 [54]. Chikungunya cases were not recorded in Indonesia for approximately 20 years, before the infection appeared to re-emerge and cause multiple outbreaks in South Sumatera, Aceh and West Java in early 2001 [48]. Since then multiple outbreaks have been reported [48, 55–65]. Despite the high number of chikungunya cases in Indonesia in recent years, comprehensive epidemiological data are lacking.

### Objectives

The overarching aim of the study was to provide a comprehensive overview of chikungunya epidemiology in Indonesia including an evolutionary analysis of CHIKV that have circulated in the country between 1983 and 2016. Through a systematic review of available literature and available sequence data on GenBank, the primary objectives of this study were: a) to estimate the incidence of chikungunya in Indonesia; b). to characterize the seroprevalence of anti-CHIKV IgM and IgG antibodies in Indonesia; c) to describe the mortality of chikungunya in Indonesia and; d) to genetically characterize CHIKV circulating in Indonesia that were isolated locally or from travelers returning from Indonesia. The secondary objective was to provide data of travel-associated chikungunya originating from Indonesia.

## Methods

### Protocol and registration

The systematic review was conducted as recommended by the Preferred Reporting Items for Systematic Reviews and Meta-analyses (PRISMA) guidelines [66]. The protocol of the systematic review was registered at PROSPERO, an international database of prospectively registered systematic reviews at the University of York (CRD42017078205).

### Role of the funding source

The funders of the study had no role in study design, data collection, data analysis, data interpretation, writing of the report and decision to publish.

### Eligibility criteria

In this study, all full articles or abstracts published in any year and language were included while editorials, reviews, commentaries, and qualitative studies were excluded for all outcomes except for mortality. This is because study reporting chikungunya mortality is limited. Studies in any setting (outbreak or non-outbreak) with any study design (cross-sectional or prospective) were considered eligible.

#### Chikungunya incidence

Any publications that reported the number of acute chikungunya infections or seroconversions over any time interval were eligible, as proposed previously [67]. Studies reporting chikungunya outbreaks with an attack rate were also considerate eligible if information about the population at risk was given.

#### Seroprevalence of anti-CHIKV IgM and IgG antibodies

Any publications that provided primary data on the seroprevalence of anti-CHIKV IgM and IgG antibodies in Indonesia were considered eligible. Eligible studies included those reporting anti-DENV IgM and/or IgG seroprevalence, measured by the presence of IgM and IgG antibodies, among individuals with suspected acute chikungunya infection, with suspected other arbovirus infection, with undifferentiated acute febrile illness or who were healthy. Studies reporting anti-DENV IgM and/or IgG during outbreak and non-outbreak settings were considered eligible.

#### Chikungunya mortality

Studies reporting chikungunya-associated deaths either during an outbreak or in a non-outbreak setting were considered eligible. For mortality, all type of articles (full papers, case studies, letters, editorials, and commentaries) with adequate information were considered eligible. Studies reporting chikungunya-associated death among travellers returning from Indonesia were excluded.

#### Chikungunya virus genotype

All CHIKV sequences originating from Indonesia, either isolated locally or in travellers returning from Indonesia, were included. CHIKV sequences originating from Indonesian mosquito samples were also included. Single and partial gene sequences, of either non-structural or structural genes, as well as complete genome sequences were included. Studies reporting CHIKV genotype information, that failed to deposit sequence data into GenBank, were excluded.

#### Exported chikungunya

All chikungunya cases reported in travellers returning from Indonesia were included. Cases were confirmed by anti-CHIKV IgM, reverse transcription polymerase chain reaction (RT-PCR) or viral isolation. Cases without laboratory confirmation were excluded.

### Information sources and search strategy

A systematic search was conducted using four bibliographical databases (EMBASE, MEDLINE, PubMed and Web of Science as of October 16th 2017) to identify potential articles using Medical Subject Headings (MeSH) encompassing the terms “Chikungunya” AND “Indonesia”. No limit was set for the publication year or language. In addition, grey literature and official reports from the MoH, published in either Indonesian or English, were included. Reference lists of the identified articles and references of deposited sequences in GenBank were also searched manually to find additional potential studies. Some authors were contacted to obtain or clarify information.

### Study selection

All titles and abstracts of identified articles were imported into the local library of EndNote X7 (Thompson Reuters, Philadelphia, PA, USA) and duplicate records between multiple databases were removed. Retrieved articles were initially screened based on title and abstract to identify possible eligible studies. The full texts of potential eligible articles were then reviewed. The screening and review process was conducted by two authors (HH and AM). After reviewing full texts, the eligibility of each study was decided. If there was disagreement between two authors, a third made the final decision.

### Data collection process and synthesis

#### Chikungunya incidence

The number of new chikungunya cases and population at risk or overall attack rate during an outbreak were recorded. Location, date, setting of study, diagnosis method, number of confirmed or suspected cases and other related information were also extracted. The incidence of chikungunya was expressed in terms of person-year.

Besides systematically reviewing the available literature, we also accessed and extracted data from the National Disease Surveillance run by the Directorate General of Disease Prevention and Control of the Indonesian MoH. Chikungunya is a notifiable disease in Indonesia. The chikungunya case definition and case ascertainment used in this surveillance system have previously been published at the National Guideline for Prevention and Control of Chikungunya from MoH [68]. This case definition follow the World Health Organization (WHO) criteria [69]. In brief, chikungunya cases were classified into three categories: a) possible case, diagnosed based on clinical criteria alone as acute onset of fever > 38.5°C and severe arthralgia/arthritis not explained by other medical conditions; b) probable case, diagnosed based on the clinical criteria as mentioned and epidemiological criteria (residing or having visited epidemic areas) and; c) confirmed case, diagnosed based on laboratory criteria which show a positive result for virus isolation, RT-PCR, IgM antibodies or a four-fold increase in IgG antibodies [68]. This surveillance system includes all types of chikungunya cases. The number of chikungunya cases were retrieved from the database. This primary dataset was analysed separately from other studies from systematic review. The annual chikungunya incidence rate was determined by dividing the number of new chikungunya cases, by the size of the population at risk based on MoH data. The incidence rate was expressed as per 100,000 person-years. To obtain a more comprehensive distribution of the chikungunya cases in Indonesia, geographical maps of provincial incidence rates from 2008 to 2016 were created using ArcGIS software [70]. The provincial incidence rate was expressed as the number of chikungunya cases per 100,000 civilians.

#### Seroprevalence of anti-CHIKV IgM and IgG antibodies

Because each study had different settings, designs and tested sera (collected from febrile or non-febrile subjects), which may potentially affect the percentage of prevalence, the seroprevalence in this study was stratified. The seroprevalence of anti-CHIKV IgM and IgG antibodies were divided into: a) setting of study (outbreak and non-outbreak) and; b) status of tested sera (collected from healthy individuals and patients with undifferentiated acute febrile illness or suspected arbovirus infection). Therefore, the seroprevalence of anti-CHIKV IgM and IgG antibodies were stratified into four categories: a) among febrile patients during a chikungunya outbreak or post outbreak; b) among non-febrile patients during a chikungunya outbreak or post outbreak; c) among febrile patients not in an outbreak; and d) among non-febrile patients not in an outbreak. If one reference consisted of two or more studies with different settings or statuses of participants, then the data were divided into the relevant categories. The median value and the ranges of the seroprevalence of IgM and IgG antibodies were calculated for each setting.

#### Chikungunya mortality

For mortality, the number of deaths associated with CHIKV infections either during an outbreak or in a non-outbreak setting was included. Data such as location, date, setting of study, sample size, diagnosis method, number of cases and other related information were extracted. The case fatality rate was calculated as the number of deaths associated with chikungunya, confirmed by doctor resume on death certificate during passive surveillance or confirmation by outbreak investigation staff run by MoH, divided by the number of cases, expressed as a percentage (%). All deaths associated with chikungunya are reported continuously in the National Disease Surveillance managed by MoH.

#### Chikungunya virus genotype

To determine the CHIKV genotype, CHIKV sequences derived from locals and travellers returning from Indonesia were included. In addition, a search was conducted in GenBank to confirm the findings from systematic review and to obtain additional sequence data. To avoid missing sequences from our search on GenBank especially from travel-related cases, for example unlabelled origin of sequences, intensive manual search was conducted on references from the systematic review. Then manual searches were conducted in GenBank. Authors were contacted if there was disagreement between the information in the article and with the GenBank data. To provide more comprehensive data of circulating genotype over time, data from a study that consisted of genotype data over multiple years was divided and reported per year. Sequences were extracted and imported to Geneious v.10.1.3 [71] for further phylogenetic analysis.

#### Exported chikungunya

To fully assess epidemiology data of chikungunya in Indonesia, all confirmed CHIKV infections (as defined by serology or RT-PCR) reported from other countries that are known to have originated from Indonesia were also included. Essential data such as date, country, and number of cases, diagnosis method, and the genotype of CHIKV were collected. Data from a study that consisted of genotype data over multiple years were also divided and reported per year, if possible.

### Outcomes

The primary outcomes of the systematic review, together with MoH database analysis, were: a) the incidence of chikungunya in Indonesia; b) the seroprevalence of anti-CHIKV IgM and IgG antibodies in Indonesia; c) chikungunya-related mortality in Indonesia and; d) a description of the CHIKV genotypes circulating in Indonesia. The secondary outcome was chikungunya cases exported from Indonesia to other countries.

### Risk of bias assessment

The quality of eligible studies of incidence and seroprevalence IgM and IgG was evaluated by evaluating the risk of bias and the precision of the reported measures in accordance with a modified tool from previous study [67]. The risk of bias was evaluated by critically appraising diagnostic methods, sampling, response rate, and study setting. Studies were considered to have a low, high, or unclear risk of bias based on those four domains. Risk of bias was considered low if: a) seroprevalence of CHIKV-specific antibodies was measured using a standard method such as enzyme-linked immunosorbent assay (ELISA), HI, and PRNT-based assays and chikungunya was diagnosed with laboratory confirmation such as IgM, RT-PCR or viral isolation; b) samples was selected using probability-based strategy - for incidence studies that enrolled individuals presenting to a health facility with acute infection this domain was not assessed; c) response rate was ≥80% and; d) the study setting was clearly stated (during outbreak or not). Studies were considered to have high precision if the number of individuals recruited or tested was ≥100 [67]. For genotyping studies, the quality of study was judged based on: a) whether the origin of sequences is clearly mentioned (human or mosquito) and; b) whether the location and date of isolation are clearly provided. The quality of studies about exported chikungunya was assessed based on two criteria: a) clear information of travel history and; b) chikungunya cases were diagnosed using standard methods such as IgM, RT-PCR or viral isolation. If at least one criterion was missing in studies on genotyping or exported chikungunya, then the study was considered to have a high risk of bias. If a reference consists more than one studies, the risk of bias was assessed for each study.

### Phylogenetic and evolutionary analysis

To provide solid data on CHIKV circulating in Indonesia, phylogenetic and evolutionary analyses were conducted for all Indonesian CHIKV sequences. To achieve this, Indonesian CHIKV sequences were downloaded from the GenBank database using the search keywords: “Chikungunya” AND “Indonesia” (last searched: October 20th, 2017). For comparison, all CHIKV sequences from other countries were also downloaded and those full genome sequences were trimmed leaving only the E1 gene. All Indonesian and comparison sequences were aligned using a Multiple Alignment using Fast Fourier Transform (MAFFT) v.7.309 [72] as implemented in Geneious v.10.1.3 [71]. To provide the relationship between Indonesian viruses and viruses isolated from other parts of world, an estimation of the maximum likelihood-based phylogenetic tree was performed using Randomized Axelerated Maximum Likelihood (RAxML) v.7.2.8 [73, 74]. General Time Reversible with gamma substitution model (GTR + Γ) and a rapid bootstrap procedure were employed with 1589 comparison sequences.

To provide evolutionary information of Indonesian viruses, their evolutionary rate and the Time to Most Recent Common Ancestors (TMRCA) was estimated using the Bayesian Markov chain Monte Carlo (MCMC) method as implemented in Bayesian Evolutionary Analysis Sampling Trees (BEAST) v.2.4.6 [75]. The input file for BEAST was prepared using Bayesian Evolutionary Analysis Utility (BEAUti) and runs were performed using General Time Reversible with four gamma category and invariant sites (GTR + Γ4 + I). The runs used the relaxed lognormal molecular clock with an initial estimated evolutionary rate of 4.33 × 10^− 4^ substitutions per site per year [76] and with a Coalescent Bayesian skyline model. A hundred million chains were run and sampled every thousand cycles with 53 representative reference sequences. The output of MCMC runs was analyzed using Tracer v.1.6 to assess effective sampling size. The Maximum Clade Credibility (MCC) of phylogenetic trees was created using TreeAnnotator v.2.4.6 with 10% burn-in and visualized using FigTree v.1.4.3.

## Results

### Study eligibility results

**Fig. 1:**
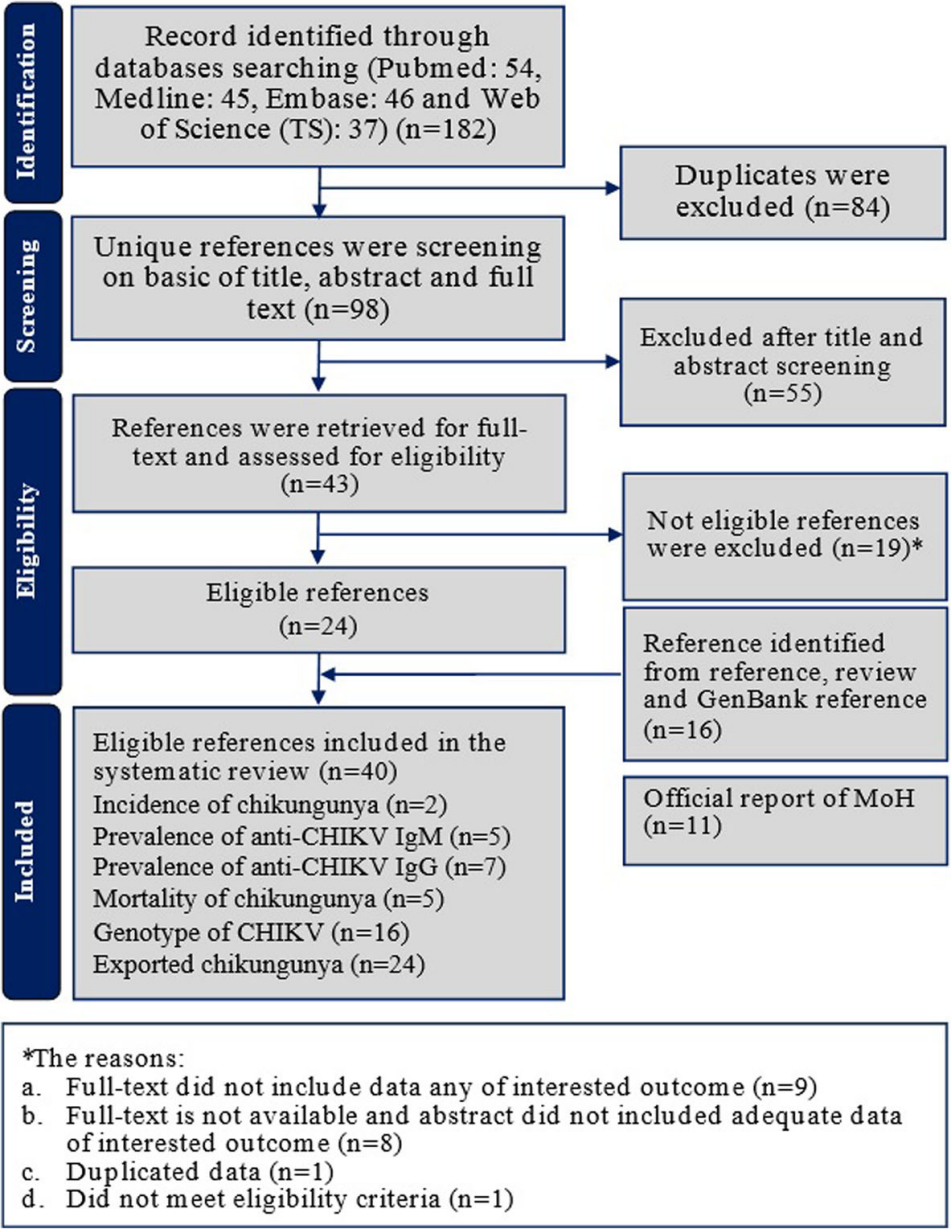
Flowchart of the result of literature search according to the preferred reporting items of systematic reviews and meta-analyses (PRISMA)

The searches yielded 182 references of which 84 were excluded as duplicates. After conducting a title and abstract screen of 98 references, an additional 55 references were excluded (Fig. 1). The full text references were retrieved for 43 titles; 19 had been excluded for one of four reasons: a) the full-text did not include data of any outcome of interest (*n* = 9); b) full-texts were not available and the abstract did not include adequate data for any outcome of interest (*n* = 8); c) duplicated data in another study (*n* = 1); and d) did not meet eligibility criteria (n = 1). This screen resulted in 24 eligible studies [13, 49, 50, 54, 55, 77–95]. An additional 16 references were identified from reference lists, reviews and GenBank references [20, 23, 76, 96–108] in which some of the references reported more than one of the outcomes of interest. A total of 40 references were included in the systematic review [13, 20, 23, 49, 50, 54, 55, 76–108]. Of these references, two studies assessed the incidence of chikungunya [94, 95], five studies assessed the seroprevalence of anti-CHIKV IgM antibodies [54, 55, 77, 78, 95] and seven assessed the seroprevalence of anti-CHIKV IgG antibodies [49, 50, 54, 55, 77–79]. Five studies reported mortality rates of chikungunya [13, 54, 55, 78, 80]. Genotype analyses of CHIKV circulating in Indonesia were reported in sixteen studies [13, 20, 23, 76, 78, 81–85, 94, 98, 100, 102, 103, 108], and exported chikungunya or travel-related chikungunya were reported in 24 studies [20, 23, 82, 84, 86–93, 96–107]. Detailed characteristics of each study is provided in Additional file 1. In addition, eleven official reports from the MoH of Indonesia, all reporting the incidence and mortality of chikungunya, were identified [48, 56–65].

### Precision and risk of bias assessment

Quality assessment for each study included on chikungunya incidence and seroprevalence anti-CHIKV IgM and IgG is provided in Table 1. Both chikungunya incidence studies, four out five (75%) and five out seven (71.4%) of references of seroprevalence anti-CHIKV IgM and IgG, respectively, contained high precision as defined by a sample size of > 100 subjects. For the risk of bias assessment, response rate was not reported in all studies and all studies used valid essay. Among seroprevalence studies, 16% (2/12) studies used probability sampling (i.e. low risk of bias) and all studies had clear setting of the study. There was no variability of risk of bias among studies on CHIKV genotype in Indonesia and exported chikungunya cases. All studies on CHIKV genotypes provided clear information of sequences (origin of the sequences, location and date of isolation) and all exported chikungunya studies had robust methods in diagnosing chikungunya and clear information of travel history (i.e. low risk of bias). See Additional file 1 for detailed risk of bias assessment for each study.

**Table 1:** Precision and risk of bias assessment of studies on chikungunya incidence and seroprevalence of anti-Chikungunya virus in Indonesia

Year	Region	Sample	Risk of bias				Precision	Reference
			Assay	Sampling	Setting	Response rate		
Chikungunya incidence studies								
2000–2004	Bandung	5704	Low	NA	Low	Unknown	High	[94]
2010–2011	Jakarta, West Java, Bali	446	Low	NA	Low	Unknown	High	[95]
Seroprevalence anti-CHIKV IgM studies								
2015–2016	Bali	15	Low	NA	Low	Unknown	Low	[78]
2004–2005	Indonesia	198	Low	NA	Low	Unknown	High	[77]
2010	Jakarta, West Java, Bali	105	Low	NA	Low	Unknown	High	[95]
2001	Bogor	99	Low	Low	Low	Unknown	Low	[55]
1998–1999	Yogyakarta	76	Low	Unknown	Low	Unknown	Low	[54]
Seroprevalence anti-CHIKV IgG studies								
2015	Bali	8	Low	NA	Low	Unknown	Low	[78]
2004–2005	Indonesia	198	Low	NA	Low	Unknown	High	[77]
1995–1996	Semarang	60	Low	NA	Low	Unknown	Low	[97]
1998–1999	Yogyakarta	76	Low	Unknown	Low	Unknown	Low	[54]
2002	Bekasi	145	Low	Low	Low	Unknown	High	[55]
1972	Ambon	321	Low	Unknown	Low	Unknown	High	[50]
1972	Kalimantan	199	Low	Unknown	Low	Unknown	High	[49]

*NA* Not applicable

### Incidence of chikungunya

Two studies assessed the incidence of chikungunya in Indonesia [94, 95]. One study took place in Bandung (West Java province) between 2000 and 2004 and between 2006 and 2008 [94]. This study found that the incidence of chikungunya was 10.1/1000 persons-year [94]. Another study in three localities in Indonesia found similar incidence, 8.8/1000 persons-year during 2010–2011 [95]. In addition, eleven annual reports from the MoH of Indonesia were identified [48, 56–65], providing the incidence rate of chikungunya between 2004 and 2015. This data revealed that the lowest chikungunya incidence was in 2005 with 0.16/100,000 person-year [48] while the highest incidence rate was recorded in 2009 with 36.2 cases per 100,000 persons-year [62]. In 2009, more than 83 thousand cases were reported and CHIKV been reported circulate in seventeen out of 34 provinces in Indonesia [62].

**Fig. 2:**
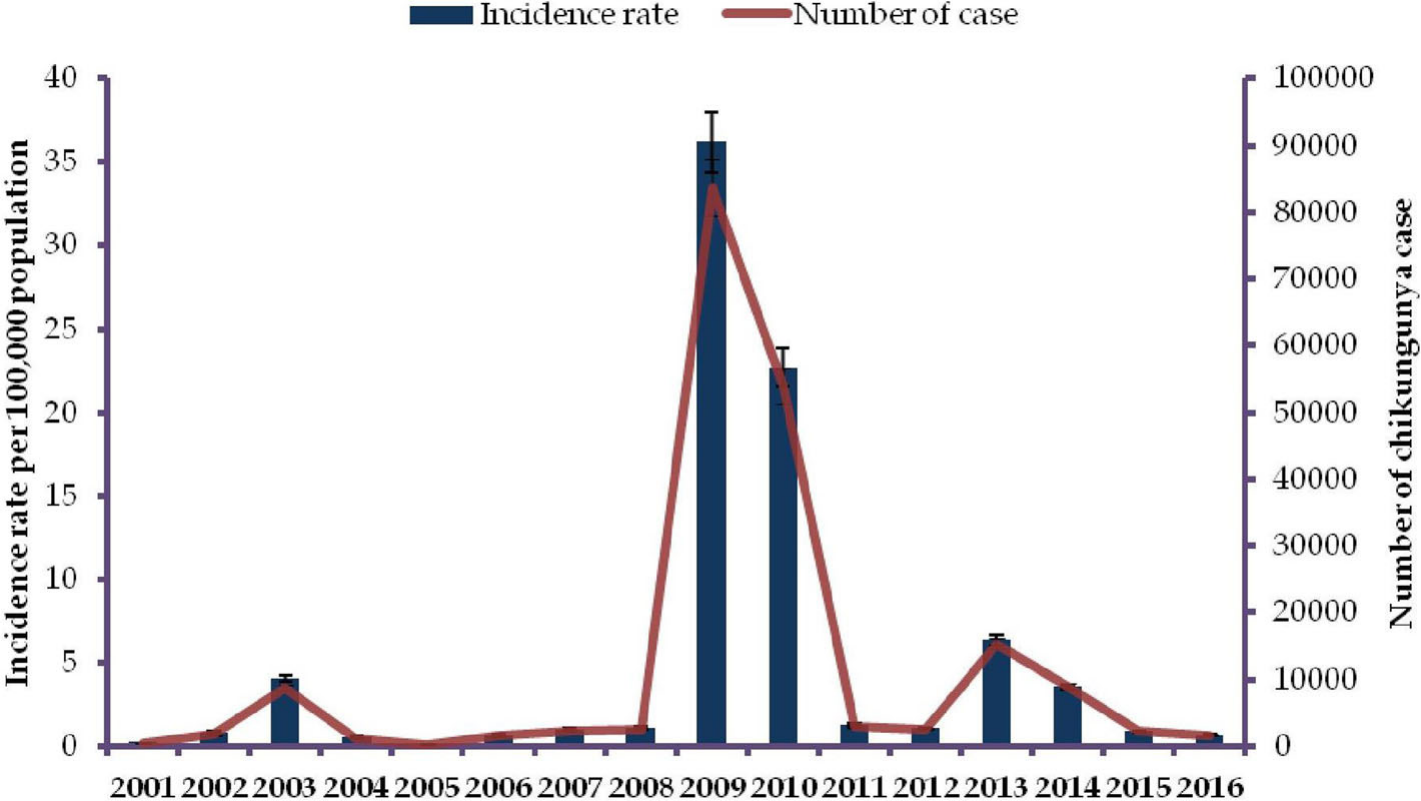
Trends of number of chikungunya cases and incidence rates of chikungunya case (per 100,000 person years) reported to the Ministry of Health of the Republic of Indonesia from 2001 to 2016

**Fig. 3:**
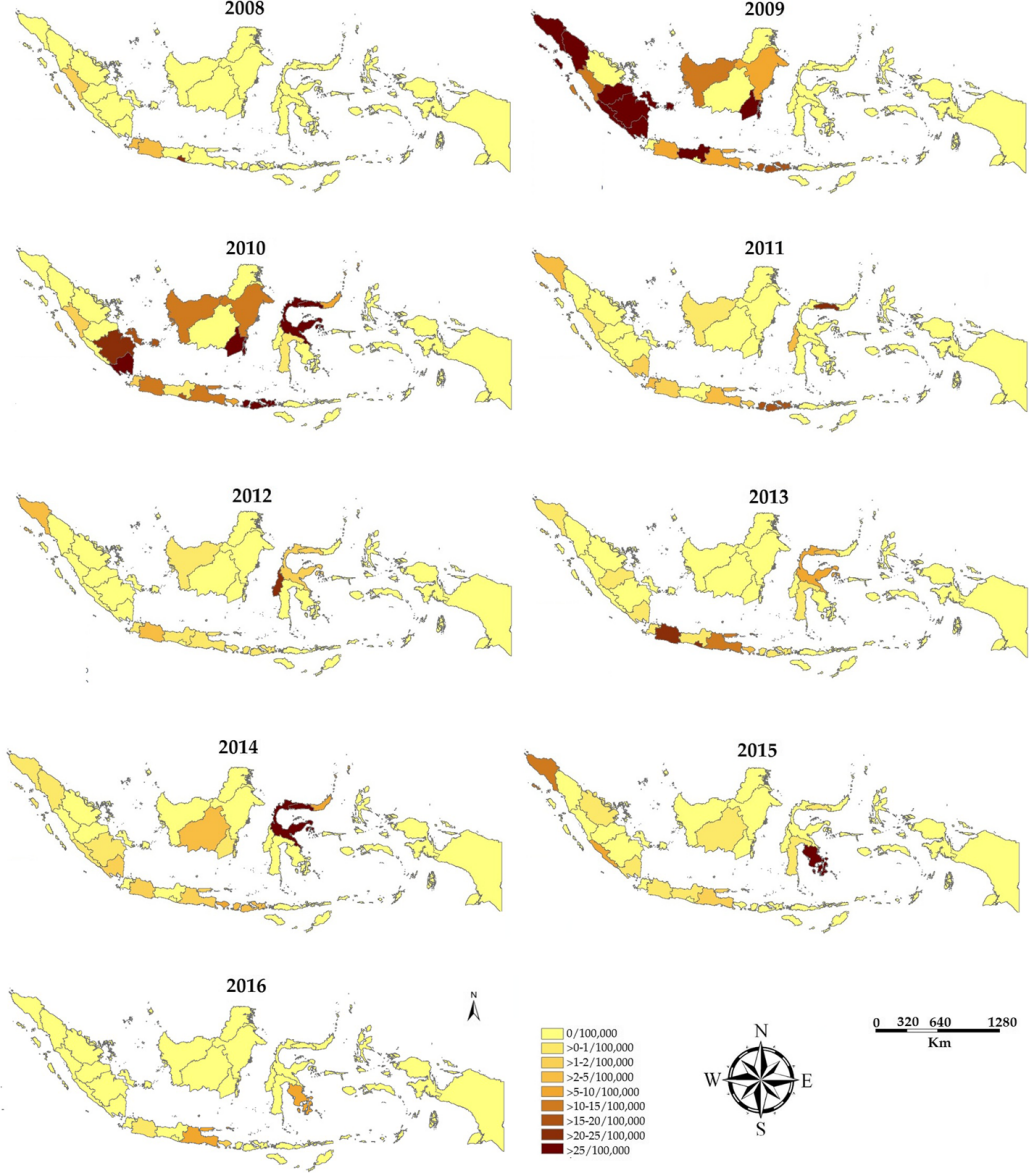
Geographical mapping of incidence rates of Chikungunya virus infection in Indonesian provinces from 2008 to 2016 (per 100,000 persons). Annual number of chikungunya cases from each Indonesian province was extracted from the Ministry of Health of the Republic Indonesia and the map was created using ArcGIS. The colour gradation indicates the incidence rates

To provide a more comprehensive picture of incidence rate of chikungunya, we accessed and extracted the National Disease Surveillance database at the MoH. The database covered chikungunya incidence between 2001 and 2016. In this 16-year period, the highest number of chikungunya cases was recorded in 2009 with 83,756 cases, equivalent to an incidence rate of 36.2 cases per 100,000 person-year (Fig. 2). The map of provinvial indicence rates indicated that chikungunya cases did not distribute equally among Indonesian regions (Fig. 3). The highest incidence of chikungunya occurred in Sumatera, Kalimantan and Java. Chikungunya was not reported in Papua and West Papua province of Indonesia between 2008 and 2016.

### Seroprevalence of anti-CHIKV IgM antibodies

Five studies were identified that assessed the seroprevalence of anti-CHIKV IgM antibodies (with or without the combination of other tests), during both outbreak and non-outbreak settings [54, 55, 77, 78, 95]. These studies analyzed a total of 1183 serum samples between 1998 and 2010. The combination of all studies that were conducted in outbreak and non-outbreak settings yielded a median IgM seroprevalence of 13.3% (range 0.0–60.4%) (Table 2).

**Table 2:** Seroprevalence of anti-Chikungunya virus IgM antibodies within certain Indonesian location as reported in published papers

Setting	Year	Region	Sample tested	Positive cases		Diagnosis method	Reference
				Number	Percentage		
Non-outbreak and febrile	2010	Jakarta, West Java, Bali	105	26	24.8	IgM ELISA	[95]
	2004–2005	Indonesia	198	7	3.5	IgM ELISA	[77]
	1998–1999	Yogyakarta	57	15	26.3	IgM ELISA	[54]
Non-outbreak and non-febrile	2002	Bekasi	55	0	0.0	IgM ELISA	[55]
	2001	Bogor	99	9	9.1	IgM ELISA	[55]
	1998–1999	Yogyakarta	123	7	5.6	IgM ELISA	[54]
Median (Non-outbreak)	1998–2010	Indonesia			7.3 (0.0–26.3)	IgM ELISA	Present study
Outbreak and febrile	2015–2016	Bali	15	2	13.3	IgM ELISA	[78]
	2002	Bekasi	93	40	43.0	IgM ELISA	[55]
	2001	Bogor	86	52	60.4	IgM ELISA	[55]
	1998–1999	Yogyakarta	61	2	3.2	IgM ELISA	[54]
Outbreak and non-febrile	2002	Bekasi	124	13	10.4	IgM ELISA	[55]
	2002	Bekasi	21	4	19.0	IgM ELISA	[55]
	2001	Bogor	45	8	17.7	IgM ELISA	[55]
	2001	Bogor	25	12	48.0	IgM ELISA	[55]
	1998–1999	Yogyakarta	76	3	3.9	IgM ELISA	[54]
Median (Outbreak)	1998–2010	Indonesia			17.7 (3.2–60.4)	IgM ELISA	Present study
Median (Total)	1998–2010	Indonesia			13.3 (0.0–60.4)	IgM ELISA	Present study

*ELISA* Enzyme-linked immunosorbent assay

Our data indicate that the seroprevalence of anti-CHIKV IgM antibodies during an outbreak is much higher compared to in a non-outbreak setting (median 17.7%, range 3.2–60.4% vs. median 7.3%, range 0.0–26.3%, respectively). In some outbreaks the seroprevalence of IgM antibodies was more than 50%, such as the outbreak in Bogor 2001 [55]. However, an investigation conducted 3 months after a chikungunya outbreak in Yogyakarta during 1998–1999 found that IgM antibodies were only present in 3.2% of suspected patients with CHIKV infection while IgG seroprevalence was at 70.4% [54] (Table 2).

### Seroprevalence of anti-CHIKV IgG antibodies

Seven studies provided information on the seroprevalence of anti-CHIKV IgG antibodies in humans, in both outbreak and non-outbreak settings [49, 50, 54, 55, 77–79]. These studies covered a 47-year period (between 1969 and 2015) with a total of 3717 serum samples tested (Table 3). Overall, in both outbreak and non-outbreak settings, the median seroprevalence of IgG antibodies was 18.5% (ranging from 0.0% in non-febrile inhabitants in non-outbreak area to 73.1% among patients who were febrile during an outbreak). In a non-outbreak setting, for both febrile and non-febrile patients, the median seroprevalence of anti-CHIKV IgG was 14.1% (range 0.0–43.9%). In contrast, the seroprevalence of IgG antibodies was much higher among patients during an outbreak (both febrile and non-febrile individuals) (median 47.4%, range 0.0–73.1%). The median seroprevalence of anti-CHIKV IgG among non-febrile subjects in a non-outbreak setting was 12.2% (range 0.0 to 26.8%) in Indonesia.

**Table 3:** Seroprevalence of anti-Chikungunya virus IgG antibodies within certain Indonesian location as reported in published papers

Setting	Year	Region	Sample tested	Positive cases		Diagnosis method	Reference
				Number	%		
Non-outbreak and febrile	2004–2005	Indonesia	198	87	43.9	IgG	[77]
	1995–1996	Semarang	60	14	23.3	IgG	[79]
	1998–1999	Yogyakarta	57	24	42.1	IgG	[54]
Non-outbreak and non-febrile	1998–1999	Yogyakarta	123	33	26.8	IgG	[55]
	2002	Bekasi	55	3	5.4	IgG	[55]
	2001	Bogor	99	13	13.1	IgG	[55]
	1972	Kalimantan	692	151	21.8	HI	[50]
	1972	Java	54	0	0.0	HI	[51]
	1972	Bali	107	2	1.9	HI	[51]
	1972	Lombok	140	6	4.3	HI	[51]
	1972	Kupang	121	15	12.4	HI	[51]
	1972	Sulawesi	298	55	18.4	HI	[51]
	1972	Ambon	321	37	11.5	HI	[51]
	1972	Kalimantan	199	28	14.1	PRNT	[50]
	1971	Sulawesi	125	24	19.2	PRNT	[50]
	1971	Ambon	64	2	3.1	PRNT	[50]
	1969–1970	Papua	243	45	18.5	PRNT	[50]
Median (Non-outbreak)	1969–2005	Indonesia			14.1 (0.0–43.9)		Present study
Outbreak and febrile	2015	Bali	8	0	0.0	IgG	[79]
	2002–2003	North Sulawesi	222	18	8.1	IgG	[56]
	2002	Bekasi	93	68	73.1	IgG	[56]
	2001	Bogor	86	58	67.4	IgG	[56]
	1998–1999	Yogyakarta	61	43	70.4	IgG	[55]
Outbreak and non-febrile	2002	Bekasi	145	65	44.8	IgG	[56]
	2001	Bogor	70	35	50.0	IgG	[56]
	1998–1999	Yogyakarta	76	34	44.5	IgG	[55]
Median (Outbreak)	1998–2015	Indonesia			47.4 (0.0–73.1)		Present study
Median (Total)	1969–2015	Indonesia			18.5 (0.0–73.1)		Present study

*HI* Haemagglutination inhibition assay, *IgG* Immunoglobulin G, *PRNT* Plaque reduction neutralization tests

### Mortality of chikungunya in Indonesia

Five outbreak investigations [13, 54, 55, 78, 80] were identified and no deaths due to chikungunya were reported. In addition, in eleven annual reports from the MoH of Indonesia, covering 44 years (1973 to 2016), there was no deaths related to CHIKV infection [48, 56–65].

### Genotypes of CHIKV circulating in Indonesia

Sixteen studies reporting the genotype of circulating Indonesian CHIKV were identified with a total of 130 viruses sequences [13, 20, 23, 76, 78, 81–85, 94, 98, 100, 102, 103, 108] (Table 4). Of these studies, seven were conducted among local inhabitants [13, 78, 81, 83, 85, 94, 108], and eight were conducted with viruses isolated from travellers returning from Indonesia [20, 23, 82, 84, 98, 100, 102, 103]. One study did not mention whether the virus was isolated in local inhabitant or in a traveller [76]. Of the seven local studies, four were conducted in a non-outbreak setting [81, 83, 85, 94], two investigations were carried out during a chikungunya outbreak [13, 78] and one study did not specify the setting [108]. Most of the viruses that were isolated from travellers were from Taiwan [23, 82, 100]. Other viruses were collected from travellers returning to Singapore [20], France [98], the Netherlands [102], Russia [84], and Germany [103]. A vast majority of the 130 CHIKV isolated from Indonesia, belonged to Asian genotype (92.3%, 120/130). The remaining isolated viruses were identified as being viruses of the ECSA genotype (7.7%, 10/130). Of these ECSA viruses, two were isolated locally in 2011 [13] and the remaining eight were isolated from travelers returning from Indonesia between 2008 and 2010 [20, 23, 102]. Viruses of the West African genotype have not yet been identified in or from Indonesia.

**Table 4:** Chikungunya virus genotypes circulating in Indonesia

Year	Location	Setting	Number of sequences	Genotype (n)	Accession number (GenBank)	Reference
2015	Jambi	Non-outbreak	8	Asian	KX097981-KX097988	[86]
2016	Bali	Outbreak	6	Asian	KY885022-KY885027	[79]
2013	Indonesia	Exported case	1	Asian	KF872195	[85]
2012	Bali	Exported case	1	Asian	KM673291	[104]
2011	Various places	Outbreak	28	ECSA (2), Asian (26)	KJ729829-KJ729856	[13]
2011	Surabaya	*Ae. aegypti* male	2	Asian	AB678689-AB678690	[84]
2010–2011	Surabaya	Non-outbreak	17	Asian	AB678691-AB678695 AB678678-AB678688	[84]
2010	Indonesia	Exported case	1	ECSA	KC862329	[103]
2009	Indonesia	Exported case	1	Asian	FR846307	[99]
2007-2014^a^	Indonesia	Exported case	29	ECSA (6), Asian (23)	KU561427-KU561432 and KU561436-KU561458	[23]
2008	Indonesia	Exported case	2	ECSA (1), Asian (1)	FJ445483, FJ445472	[20]
2007	Indonesia	Exported case	1	Asian	EU192143	[83]
2007–2008	Indonesia	Exported case	7	Asian	FJ807897, FJ807886-FJ807891	[101]
2007^b^	Bandung	Non-outbreak	3	Asian	KT175539-KT175541	[82]
2000–2008	Bandung	Non-outbreak	20	Asian	KC879559-KC879578	[95]
1985	Indonesia	Unknown	1	Asian	HM045797	[77]
1985	Ambon	Unknown	1	Asian	AF192894	[109]
1983	Indonesia	Unknown	1	Asian	HM045791	[77]

*ECSA* East-Central South African gebnotype

^a^ ECSA genotype was isolated during 2009–2010 only

^b^ Sequences are part of non-structural gene

To confirm the results of the search from systematic review for CHIKV genotype literature and the database, a search was conducted in GenBank which yielded 119 sequences, rather than the expected 130 sequences. Discrepancy between these findings is due to three reasons: a) eight sequences were from an *in press* study and had not yet been deposited into GenBank when the search was conducted [85]; b) two sequences of CHIKV, which were isolated from Singaporean travelers returning from Indonesia, were deposited into GenBank as Singaporean viruses [20] and; c) one sequence had no information of country of origin in GenBank.

**Fig. 4:**
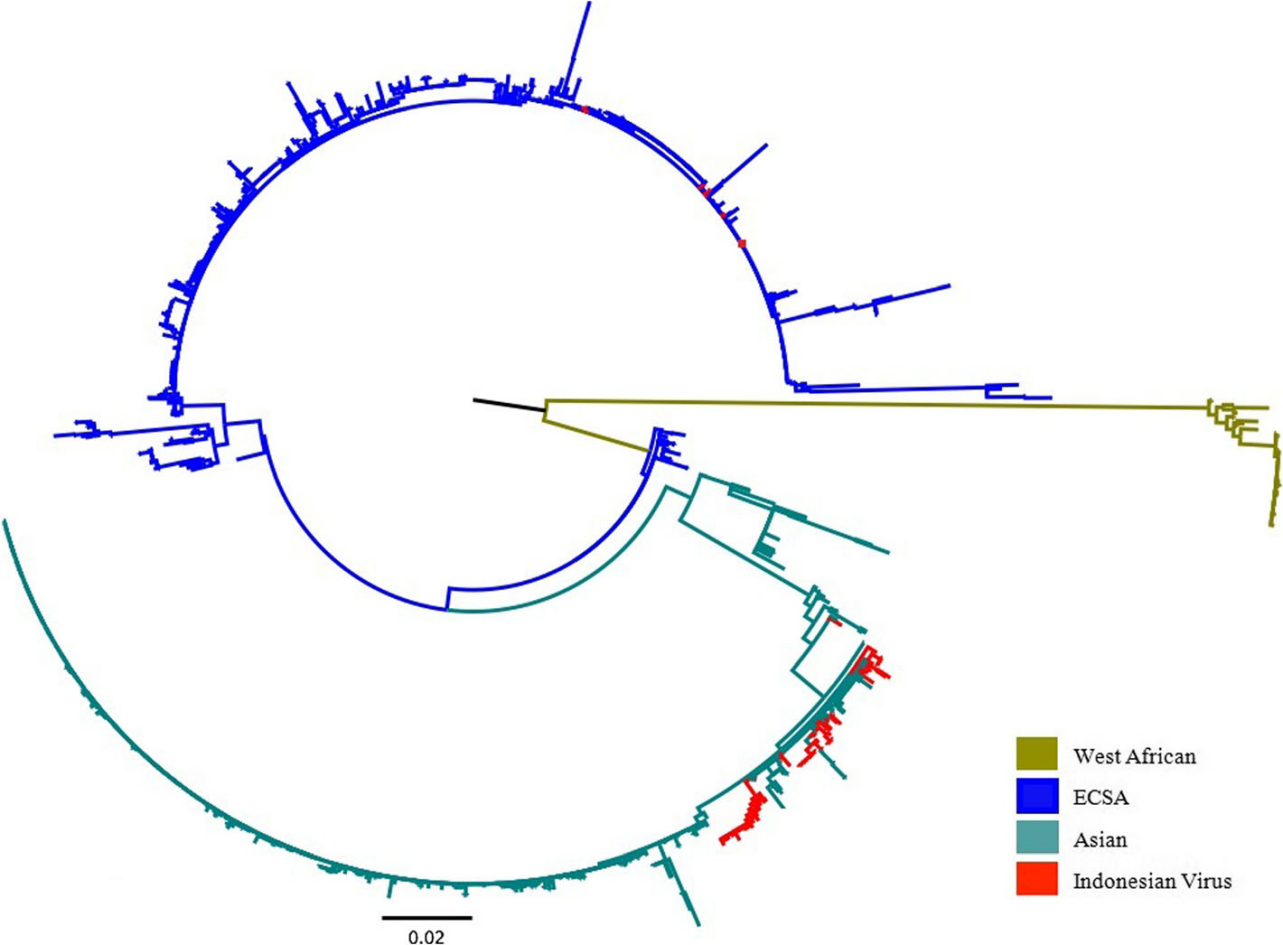
Phylogenetic tree of 127 Indonesian Chikungunya viruses and 1589 reference sequences from GenBank. The phylogenetic tree was generated using the maximum likelihood (ML) method available in the Randomized Axelerated Maximum Likelihood (RAxML) program with General Time Reversible and gamma substitution model (GTR + Γ). The tree shows the position of ten viruses of East/Central/South African (ECSA) genotype and 120 of Asian genotype from Indonesia relative to other viruses isolated worldwide

For phylogenetic and evolutionary analyses based on CHIKV E1 gene sequences, 127 sequences were included. One paper, reporting non-structural gene sequence of three Asian genotype viruses, was excluded [81]. The relationship of Indonesian CHIKV to other CHIKV isolated worldwide is shown in Fig. 4. The Asian genotype was first identified in Indonesia in 1983 [76], while viruses of the ECSA genotype were isolated for the first time in 2008, following the isolation of this genotype from returning Taiwanese [100] and Singaporean travellers [20].

**Fig. 5:**
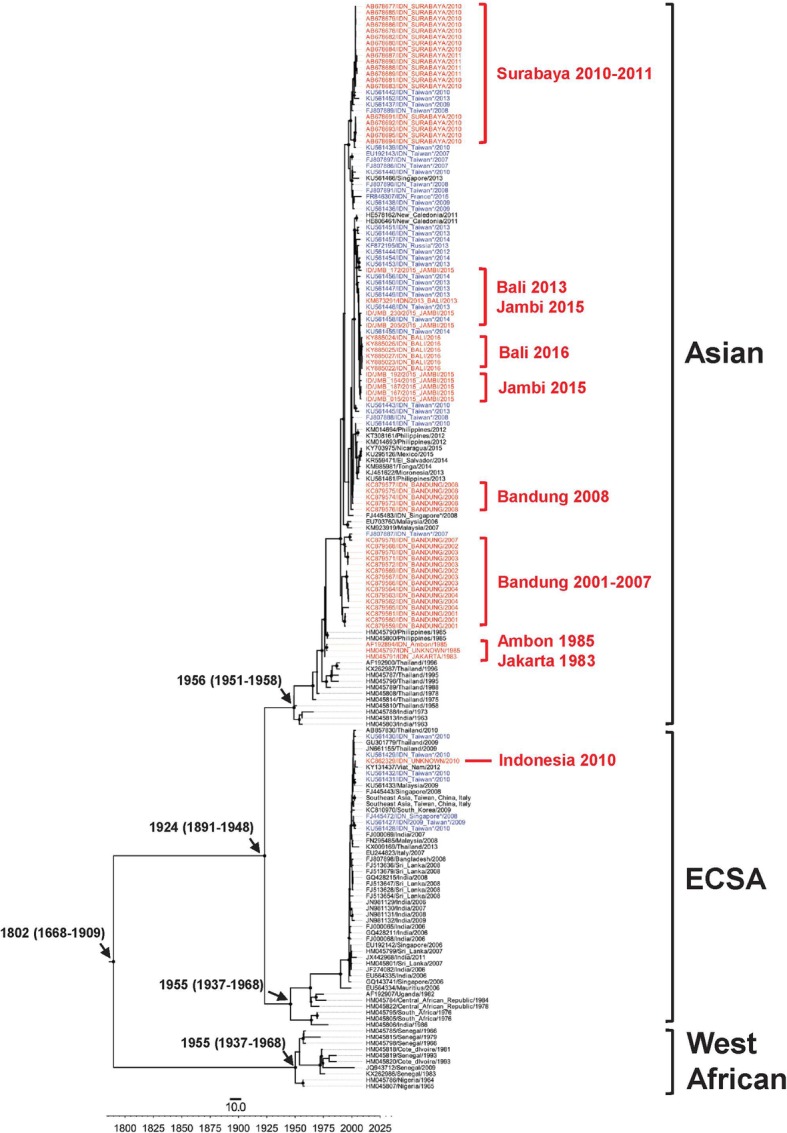
The maximum clade credibility (MCC) tree of Indonesian Chikungunya virus. The tree was generated using the Bayesian Markov chain Monte Carlo (MCMC) method as implemented in BEAST using General Time Reversible (GTR) evolution model from E1 sequences. In the analysis, 127 Indonesian chikungunya viruses and 53 representative reference sequences from GenBank were included. All Indonesian Chikungunya viruses, isolated locally (red front) or isolated in neighboring countries (blue front) are grouped into Asian and East/Central/South African (ECSA) genotype

MCMC analysis using 53 representative reference sequences reveals that the ECSA viruses sampled in Indonesia between 2008 and 2011 belong to the Indian Ocean Lineage (IOL) and are closely related to viruses that have circulated in Southeast Asian countries such as Sri Lanka, Malaysia, Singapore, Thailand, and Myanmar, as well as China and South Korea during the same period (Fig. 5). Interestingly, viruses of the ECSA genotype have not been sampled in Indonesia, either in local or from travelers studies, from 2011 onwards.

### Exported chikungunya cases

Twenty four studies, reporting exported chikungunya cases that originated from Indonesia covering a 26 year-period from 1989 to 2014, were identified [20, 23, 82, 84, 86–93, 96–107] (Table 5). During this period, a total of 195 cases of chikungunya cases were reported from travellers returning from Australia (128 cases) [86–88, 99, 104–107], Taiwan (47 cases) [23, 82, 100], Japan (4 cases) [89, 90] and other countries in Asia, Europe and the Pacific region (16 cases) [76, 84, 91–93, 96, 98, 101–103, 109]. Most of these cases were diagnosed with a combination of serology and RT-PCR, and sequence data was generated from only 27.6% (54/195) of cases [20, 23, 82, 84, 98, 100–103].

**Table 5:** Exported chikungunya cases originating from Indonesia

Year(s)	Country of reporting	Number of cases	Diagnosis method	Genotype (n)	Reference
2013–2014	Australia	47	IgM, IgG, RT-PCR and isolation	Unknown	[89]
2013	Russia	1	RT-PCR, isolation and sequencing	Asian (1)	[85]
2013	New Caledonia	1	RT-PCR and sequencing	Asian (1)	[102]
2012–2014	Taiwan	23	RT-PCR, isolation and sequencing	Asian (15) and unidentified (8)	[23]
2012–2013	Australia	34	IgM, IgG, RT-PCR and isolation	Unknown	[88]
2012	Germany	1	Isolation and sequencing	Asian (1)	[104]
2011–2012	Australia	2	IgM, IgG, RT-PCR and isolation	Unknown	[87]
2010–2011	Australia	32	IgM, IgG, RT-PCR and isolation	Unknown	[106]
2011	New Caledonia	2	Serology	Unknown	[98]
2010	Brazil	1	IgM and IgG	Unknown	[94]
2010	Brazil	1	IgM and HI test	Unknown	[97]
2010	Japan	1	IgM and RT-PCR	Unknown	[90]
2010	Netherlands	1	Isolation and sequencing	ECSA (1)	[103]
2009–2010	South Korea	3	IgM	Unknown	[92]
2009–2010	Australia	7	IgM, IgG, RT-PCR and isolation	Unknown	[105]
2009–2010	Taiwan	16	RT-PCR, isolation and sequencing	Asian (8), ECSA (6) and unidentified (2)	[23]
2009	Japan	3	IgM and PRNT	Unknown	[91]
2009	France	1	IgM, RT-PCR and sequencing	Asian (1)	[99]
2008–2009	Australia	2	IgM, IgG, RT-PCR and isolation	Unknown	[108]
2009	Singapore	3	IgM, RT-PCR and sequencing	Asian (1), ECSA (1)	[20]
2007	Australia	3	IgM, IgG and RT-PCR	Unknown	[107]
2007	Taiwan	1	RT-PCR, isolation and sequencing	Asian (1)	[83]
2006–2009	Taiwan	7	RT-PCR, isolation and sequencing	Asian (7)	[101]
2006	Europe	1	IgM, IgG or RT-PCR	Unknown	[93]
1989	Australia	1	IgM, IgG and isolation	Unknown	[100]

*ECSA East/Central/South African genotype, HI* Haemagglutination inhibition, *IgG* Immunoglobulin G, *IgM* Immunoglobulin M, *PRNT* Plaque reduction neutralizing test, *RT-PCR* Reverse transcription polymerase chain reaction

## Discussion

Our systematic review and analysis of MoH database examine the epidemiology of chikungunya in Indonesia by summarizing published studies on chikungunya incidence, the seroprevalence of IgM and IgG, mortality, CHIKV genotypes and exported cases. Existing literature on incidence rate of chikungunya Indonesia is lacking. Nevertheless, using MoH databases, we are able to generate comprehensive incidence rates of chikungunya in Indonesia over a 16 year period, from 2001 to 2016. Incidence data indicate there is no typical pattern of chikungunya in the country. The most notable finding is the massive nationwide outbreak during 2009–2010 with 137,655 cases followed by a smaller nationwide outbreak in 2013 with 15,324 cases (Fig. 2). Prior to 2008 the incidence of chikungunya was less than 10,000 cases/year and this increased significantly in 2009 and 2010 with 83,756 and 53,899 cases, respectively [61, 62] (Fig. 2) indicating that chikungunya incidence increased more than 20 times in 2009. There is no scientific evidence to explain what occurred during this outbreak. The MoH suggests that this increase was due to the fact that prior to 2009, many regions did not include CHIKV case reports in their annual reports to the MoH.

Despite the multiple outbreaks that have occurred in Indonesia since its re-emergence, there is also a lack of data regarding the magnitude of CHIKV exposure in the Indonesian population. Our systematic review, covering the period of 1998–2010, indicates that CHIKV-specific IgM antibodies were detected approximately 13% of sera collected from healthy and acute febrile illness patients (range 0.0–60.4%). This estimate can be interpreted as evidence of recent CHIKV infection. Although IgM may persist for months to years following the resolution of infection, IgM titers reduce to undetectable levels after weeks for most chikungunya patients and are replaced with long lasting IgG [4]. The high seroprevalence of anti-CHIKV IgM indicates that chikungunya is endemic in Indonesia with on-going transmission.

Interestingly, 10.8% of sera from febrile patients (presenting with dengue-symptoms or undifferentiated acute febrile illness) in a non-outbreak setting had anti-CHIKV IgM [77, 95]. This finding suggests that many patients presenting with dengue-like symptoms had a CHIKV infection. This proposed consideration is supported by the evidence from other studies: a) in a non-outbreak setting, approximately 10% of febrile patients with dengue-like symptoms demonstrated titers of anti-CHIKV IgM [110]; and b) in most Asian countries, DENV and CHIKV co-circulate in the same location [111, 112]. The confusion between dengue and chikungunya infection could explain underreporting of CHIKV in Indonesia [113]. Therefore, further tests is required to exclude CHIKV infection in patients with dengue-like symptoms. However, many regions in Indonesia lack a specific diagnostic test for chikungunya. In clinical settings in Indonesia, however, DENV infection must be considered first as the main diagnosis to minimize the incidence of severe forms of dengue and therefore to reduce dengue mortality. This rationale is based on the fact that dengue more frequently presents with severe forms compared to CHIKV, and has a high mortality rate in Indonesia [114].

We found that in both outbreak and non-outbreak localities, the median seroprevalence of asymptomatic residents with current CHIKV infection (the present of IgM) was 9.1% (range 0.0–48.0%) (Table 2). This finding highlights the important fact that not all CHIKV infections present with specific symptoms that are noticed by the infected individuals. A recent systematic review revealed that the proportion of CHIKV infections that were asymptomatic was highly variable, ranging from 3.2 to 82.1% [25]. Interestingly, current studies indicate there is an increasing trend of asymptomatic chikungunya over time [7, 21, 27]. Except for one study, all studies published from 2013 onwards reported that the percentage of asymptomatic chikungunya was more than 30% [25].

Our systematic review indicates that the median seroprevalence of CHIKV-specific IgG antibodies was 18.5% (range 0.0–73.1%) among the total population in all settings. However, from an epidemiological perspective, data from non-febrile subjects collected in a non-outbreak setting is the best representation of the true seroprevalence of CHIKV infection. The median seroprevalence of anti-CHIKV IgG in this population was 12.7% (range 0.0–26.8%) in Indonesia. This figure is higher than the estimated seroprevalence of anti-CHIKV IgG antibodies in Malaysian adults, which is at 5.9% [115]. As predicted, the median seroprevalence of IgG antibodies among healthy residents living in post-outbreak areas is much higher (approximately three times higher) compared to healthy residents in non-outbreak areas (median 44.8%, range 55.5–50.5% vs. median 12.7%, range 0.0–26.8%). This finding is comparable with other outbreak investigations, including 20.0% in Brazil (2016) [116], 26.8% in Thailand (2014) [117], 37.2% in Mayotte, Indian Ocean (2006) [118] and 67.9% in India (2007) [119]. The variability between these estimates is influenced by several factors such as serum collection time relative to outbreak, criteria of sample selection (whether asymptomatic residents during the outbreak are included or excluded) and the location of the serosurvey relative to the epicentrum of outbreak.

Surprisingly, no chikungunya-associated deaths have been recorded in Indonesia since this disease was officially recognized by MoH of Indonesia in 1973 [48]. Since 2004, chikungunya has been included in the National Diseases Surveillance run by the MoH and has been reported in the Annual Report of Indonesia Health Profile, the formal annual report from MoH of Indonesia, since 2005 [48]. Although a substantial incidence rate has been reported, no deaths have been recorded since then. In the National Guideline of Prevention and Control of Chikungunya from MoH [68], there is no clear criteria for assessing or reporting chikungunya-related deaths that should be included in the surveillance system. Unsurprisingly, this lack of guidance has led to no reported deaths from chikungunya in Indonesia; and this number is probably due to underreporting of fatal chikungunya cases within the current passive surveillance system. In fact several studies in India [120, 121], Mauritius [12] and Brazil [122, 123] during ECSA genotype outbreaks and in Dominican Republic [124] during an Asian genotype outbreak revealed an increase in the mortality rate, with deaths not adequately identified by passive surveillance systems. In addition, those studies revealed an increase of excess mortality [12, 120, 122–124] indicating that the actual proportion of chikungunya-associated deaths is underestimated in many settings. In Indonesia, during the 2008–2009 chikungunya outbreaks, there were 137,655 chikungunya cases officially reported to MoH, with no reported death. In addition, given the fact that the illnesses caused by CHIKV and DENV are clinically indistinguishable and not all reported dengue cases in Indonesia are confirmed by laboratory testing, leads to the possibility that deaths occur due to chikungunya which may be wrongly attributed to dengue as a consequence of the prioritization of dengue surveillance. Chikungunya has been included in the Annual Report of Indonesia Health Profile since 2005 [48, 56–65], but was excluded in the 2016 MoH Annual Report [125]. In the 2017 Annual Report, only 126 chikungunya cases were reported to MoH, which dropped from 1702 cases that were reported in 2016 [126]. These points reflect that chikungunya is not a priority disease in Indonesia and that there is poor chikungunya surveillance and an insufficient reporting system in Indonesia. If fact, in some studies, higher fatality rate for chikungunya was reported compared to dengue [122, 127, 128]. Therefore, the MoH should provide clear criteria for chikungunya-associated death in the national guidelines and institute a clear reporting system. In addition, in outbreaks, active surveillance might be required to reduce underreporting of chikungunya deaths in the country.

To date, two genotypes of CHIKV have been isolated in Indonesia (Asian and ECSA). Viruses of the West African genotype have not yet been isolated from Indonesia. Viruses of the Asian genotype were first isolated in Indonesia 35 years ago [76]. The ECSA genotype however was identified for the first time in 2008 [20, 100] and at the same time this genotype caused several major outbreaks in Southeast Asia countries [16, 100, 129]. This suggests that chikungunya outbreaks that occurred prior to 2008 in Indonesia were associated with viruses of the Asian genotype.

A phylogenetic analysis revealed that the ECSA viruses sampled from Indonesia during 2008–2011 are closely related with viruses that caused contemporaneous outbreaks in Southeast and East Asian countries, such as Malaysia (2008–2009) [129], Singapore (2008) [19, 20], Thailand (2008–2009) [16–18], and China (2010) [130]. This suggests that the viruses in Indonesia may have been introduced from another country in Southeast Asia, most likely Malaysia [13]. In 2008, a nationwide chikungunya outbreak occurred in Malaysia, which was found to be caused by viruses of the ECSA genotype [129]. In the same year ECSA viruses were also reported in Indonesia [20, 100] and continued to be sampled until 2011 [23]. Furthermore, our phylogenetic analysis revealed that the ECSA viruses that circulated in Southeast Asia, including Indonesia, were introduced from India and Sri Lanka, where CHIKV circulated in 2005–2007 and infected more than 1.3 million persons during 2005–2006 in India alone [14].

Our data, together with other evidence, suggest that the introduction of the ECSA genotype to Indonesia may be the reason for the large chikungunya outbreak reported between 2009 and 2010 in the country (Fig. 2). First, the Asian genotype had been the dominant genotype of Indonesia and had circulated for several decades. However, the annual number of cases never exceeded more than 10,000 cases prior to 2009. Second, the ECSA was first reported in Indonesia in 2008, and had not been isolated in the multiple studies of Indonesian CHIKV (conducted both locally and in neighboring countries) prior to this time [81, 82, 94, 100]. This evidence indicates a time relationship between the introduction of the ECSA genotype into Indonesia and a sharp increase of reported chikungunya cases. Third, within the same time-frame, nationwide outbreaks of chikungunya associated with viruses of the ECSA genotype occurred in Malaysia [129], Singapore [19] and Thailand [16]. The ECSA viruses isolated from these outbreaks are all closely related, indicating that these outbreaks were probably caused by the same type of virus that then dispersed throughout the region and likely into Indonesia as well (Fig. 5). Other scientific evidence also supports this finding of chikungunya outbreaks in Southeast Asian countries during that time-frame being driven by the emergence of the same strain of ECSA [131]. In addition, MoH data reveal that the highest number of CHIKV cases in 2009 were reported in Bangka Belitung and provinces in Sumatera Island (Jambi, South Sumatera, Lampung, Bengkulu, North Sumatera and Aceh) [62]. These provinces are in close geographical proximity to Malaysia and Singapore (Fig. 3). Finally, evolutionally analysis reveals that ECSA viruses isolated from Indonesia are closely related to those viruses associated with massive outbreaks in Malaysia, Singapore and Thailand (Fig. 5). This possibility is also supported by the fact that ECSA is more adaptive to *Ae. albopictus* due to a mutation in the envelope protein gene (E1-A226V) [132]. This mutation associated with a significant increase in viral infectivity for *Ae. albopictus*, and led to more efficient viral dissemination [132]. In the area where both *Ae. aegypti* and *Ae. albopictus* are endemic, like Indonesia, introduction of ECSA viruses could cause a massive outbreak. This evidence, in aggregate, suggests that the massive outbreaks in Indonesia during 2009 and 2010 were caused by the introduction of the ECSA genotype to the country.

There are some limitations of this study that should be discussed. First, full-texts of some potential references that assess the seroprevalence of the anti-CHIKV antibodies were not available, especially those published prior to 1985 [133–142]. Second, in the seroprevalence section of this paper, we included a study that utilized the HI test as a diagnostic method [50]. This test is quite sensitive for detecting alphavirus antibodies; however, cross-reactions often occur among the viruses of the same group. Nevertheless, a great majority of sera from this study reacted with only one of the alphaviruses by the HI test and therefore the results from that study were valid. Third, the collection time of the sera, relative to time of infection, among studies were not the same. This might influence the percentage of samples that were positive for IgM and IgG among studies. For example, an outbreak investigation in Yogyakarta reported a significantly lower of seroprevalence of anti-CHIKV IgM and a higher IgG seroprevalence compared to other studies, as the investigation was conducted after the resolution of the outbreak [54]. Fourth, in Indonesia the absence of serological surveillance means that the precise numbers of chikungunya cases reported to the MoH during outbreaks are often diagnosed clinically only. Fifth, some of the known CHIKV of the Asian genotype, that have been isolated previously, were not available in GenBank [101]. Finally, some CHIKV sequences originating from Indonesia may have been reported as deriving from elsewhere, if a returning traveller had an incomplete or inaccurate travel history. Studies may have failed to report Indonesia as the country of origin of isolated viruses, and instead, reported the country in which the virus was isolated as the origin [20]. Despite these limitations, this study is, to the best of our knowledge, the first systematic review on CHIKV infection in Indonesia. Strengths also include a search strategy that avoided missing potential references and Indonesian CHIKV sequences. In addition, this study is also able to provide the incidence rate of chikungunya for a 16-year period using the National Diseases Surveillance database from the MoH.

## Conclusion

Evidence suggests that CHIKV has circulated in most of the Indonesian archipelago for at least 50 years, despite the first virologically confirmed outbreak being recorded as recently as 1982. Although anti-CHIKV IgM and IgG seroprevalence is high and two massive nationwide outbreaks have been recorded in Indonesia, no deaths have been reported. The lack of reported deaths possibly reflects under-reporting of fatal cases in the country using the current surveillance system. Data from both locals and travelers revealed that the Asian genotype has been identified in Indonesia for more than 30 years, while viruses of the ECSA genotype were sampled only during the 2008–2011 period. A phylogenetic analysis reveals that the ECSA viruses sampled in Indonesia during this period are closely related to viruses that have circulated in neighboring Southeast Asian countries, as well as China and South Korea, within the same timeframe. It is likely that ECSA viruses were introduced to Indonesia from a neighboring country within the region, in 2008. Spatio-temporal, phylogenetic and evolutionary data suggest that this genotype was likely associated with the large 2009–2010 chikungunya outbreak in Indonesia.

## Additional file

Additional file 1: Individual characteristics and risk bias assessment of included studies in the systematic review (PDF 532 kb)

## Abbreviations

BEAST: Bayesian Evolutionary Analysis Sampling Trees
CHIKV: Chikungunya virus
DENV: Dengue virus
ECSA: East/Central/South African
ELISA: Enzyme-linked immunosorbent assay
GTR + Γ: General Time Reversible, gamma substitution model
HI: haemagglutination inhibition assay
MAFFT: Multiple Alignment using Fast Fourier Transform
MCC: Maximum Clade Credibility
MCMC: Bayesian Markov chain Monte Carlo
MoH: Ministry of Health, Republic of Indonesia
NSP: Non-structural proteins
PRISMA: Preferred reporting items for systematic reviews and meta-analyses
PRNT: Plaque reduction neutralization test
RaxML: Randomized Axelerated Maximum Likelihood
RT-PCR: Reverse transcription polymerase chain reaction
TMRCA: Time to most recent common ancestors
WHO: World Health Organization
ZIKV: Zika virus
