# Supplementary material for: Chikungunya virus infection in Indonesia: a systematic review and evolutionary analysis
Source: BMC Infect Dis. 2019 Mar 12;19:243. doi: 10.1186/s12879-019-3857-y (PMC6417237; doi:10.1186/s12879-019-3857-y)
Supplement: Supplementary file 1 — Individual characteristics and risk bias assessment of included studies in the systematic review (PDF 532 kb) [file 12879_2019_3857_MOESM1_ESM.pdf]

Additional file 2 - Individual characteristics and risk bias assessment of included studies in the systematic review

Table A. Individual characteristics of included studies on incidence of chikungunya in Indonesia

Study	Setting	Presentation	Outbreak	Year	Regions	Design	Sex ratio (M/F)	Age range	Chikungunya diagnosis	Number of cohort	Incidence rate	Risk of bias				Precision	Reference
												Assay	Sampling	Setting	Response rate		
Kosasih *et al*.	Community-based	Febrile	No	2000-2004	Bandung	Prospective	1.85: 1	18-66 years	RT-PCR, viral isolation, IgM	5,704	10.1/100 persons-year	Low	NA	Low	Unknown	High	[1]
Capeding *et al*.	Community-based	Febrile	No	2010 2011	Jakarta, West Java, Bali	Prospective	Unknown	2-14 years	NS1 positive	446	8.8 /100 person-years	Low	NA	Low	Unknown	High	[2]

Table B. Individual characteristics of included studies on seroprevalence of anti-CHIKV IgM antibodies in Indonesia

Study	Setting	Presentation	Outbreak	Year	Region	Design	Subject	Sample tested	Positive cases		Diagnosis method	Risk of bias				Precision	Reference
									Number	%		Assay	Sampling	Setting	Response rate		
Sari *et al*.	Hospital-based	Febrile	Yes	2015-2016	Bali	Cross-sectional	Chikungunya suspect patients	15	2	13.3	IgM ELISA	Low	NA	Low	Unknown	Low	[3]
Ngwe *et al*.	Hospital-based	Febrile	No	2004-2005	Indonesia	Cross-sectional	Dengue suspect patients	198	7	3.5	IgM ELISA	Low	NA	Low	Unknown	High	[4]
Capeding *et al*.	Community-based	Febrile	No	2010	Jakarta, West Java, Bali	Prospective	Febrile episodes	105	26	24.8	IgM ELISA	Low	NA	Low	Unknown	High	[2]
Laras *et al*.	Community-based	Febrile	Yes	2002	Bekasi	Cross-sectional	Chikungunya suspect patients	93	40	43.0	IgM ELISA	Low	NA	Low	Unknown	Low	[5]
Laras *et al*.	Community-based	Non-febrile	Yes	2002	Bekasi	Cross-sectional	Healthy individuals	124	13	10.4	IgM ELISA	Low	Low	Low	Unknown	High	[5]
Laras *et al*.	Community-based	Non-febrile	Yes	2002	Bekasi	Cross-sectional	Healthy individuals	21	4	19.0	IgM ELISA	Low	Low	Low	Unknown	Low	[5]
Laras *et al*.	Community-based	Non-febrile	No	2002	Bekasi	Cross-sectional	Healthy individuals	55	0	0.0	IgM ELISA	Low	Low	Low	Unknown	Low	[5]
Laras *et al*.	Community-based	Febrile	Yes	2001	Bogor	Cross-sectional	Chikungunya suspect patients	86	52	60.4	IgM ELISA	Low	NA	Low	Unknown	Low	[5]
Laras *et al*.	Community-based	Non-febrile	Yes	2001	Bogor	Cross-sectional	Healthy individuals	45	8	48.0	IgM ELISA	Low	Low	Low	Unknown	Low	[5]
Laras *et al*.	Community-based	Non-febrile	Yes	2001	Bogor	Cross-sectional	Healthy individuals	25	12	48.0	IgM ELISA	Low	Low	Low	Unknown	Low	[5]
Laras *et al*.	Community-based	Non-febrile	No	2001	Bogor	Cross-sectional	Healthy individuals	99	9	9.1	IgM ELISA	Low	Low	Low	Unknown	Low	[5]
Porter *et al*.	Community-based	Febrile	Yes	1998-1999	Yogyakarta	Cross-sectional	Were chikungunya suspect patients	61	2	3.2	IgM ELISA	Low	NA	Low	Unknown	Low	[6]
Porter *et al*.	Community-based	Non-febrile	Yes	1998-1999	Yogyakarta	Cross-sectional	Healthy individuals	76	3	3.9	IgM ELISA	Low	Unknown	Low	Unknown	Low	[6]
Porter *et al*.	Community-based	Febrile	No	1998-1999	Yogyakarta	Cross-sectional	Healthy individuals	57	15	26.3	IgM ELISA	Low	Unknown	Low	Unknown	Low	[6]
Porter *et al*.	Community-based	Non-febrile	No	1998-1999	Yogyakarta	Cross-sectional	Healthy individuals	123	7	5.6	IgM ELISA	Low	Unknown	Low	Unknown	High	[6]

Table C. Individual characteristics of included studies on seroprevalence of anti-CHIKV IgG antibodies in Indonesia

Study	Setting	Presentation	Outbreak	Year	Region	Design	Subject	Sample tested	Positive cases		Diagnosis method	Risk of bias				Precision	Reference
									Number	%		Assay	Sampling	Setting	Response rate		
Sari *et al.*	Hospital based	Febrile	Yes	2015	Bali	Cross-sectional	Chikungunya suspect patients	8	0	0.0	IgG	Low	NA	Low	Unknown	Low	[3]
Ngwe *et al.*	Hospital-based	Febrile	No	2004-2005[a]	Indonesia	Cross-sectional	Dengue suspect patients	198	87	43.9	IgG	Low	NA	Low	Unknown	High	[4]
Suharti *et al.*	Hospital-based	Febrile	No	1995-1996	Semarang	Cross-sectional	Dengue suspect patients	60	14	23.3	IgG	Low	NA	Low	Unknown	Low	[7]
Porter *et al.*	Community-based	Febrile	Yes	1998-1999	Yogyakarta	Cross-sectional	Were chikungunya suspect patients	61	43	70.4	IgG	Low	NA	Low	Unknown	Low	[6]
Porter *et al.*	Community-based	Non-febrile	Yes	1998-1999	Yogyakarta	Cross-sectional	Healthy individuals	76	34	44.5	IgG	Low	Unknown	Low	Unknown	Low	[6]
Porter *et al.*	Community-based	Febrile	No	1998-1999	Yogyakarta	Cross-sectional	Were chikungunya suspect patients	57	24	42.1	IgG	Low	NA	Low	Unknown	Low	[6]
Porter *et al.*	Community-based	Non-febrile	No	1998-1999	Yogyakarta	Cross-sectional	Healthy individuals	123	33	26.8	IgG	Low	Unknown	Low	Unknown	High	[6]
Laras *et al.*	Community-based	Febrile	Yes	2002	Bekasi	Cross-sectional	Chikungunya suspect patients	93	68	73.1	IgG	Low	NA	Low	Unknown	Low	[5]
Laras *et al.*	Community-based	Non-febrile	Yes	2002	Bekasi	Cross-sectional	Healthy individuals	145	65	44.8	IgG	Low	Low	Low	Unknown	High	[5]
Laras *et al.*	Community-based	Non-febrile	No	2002	Bekasi	Cross-sectional	Healthy individuals	55	3	5.4	IgG	Low	Low	Low	Unknown	Low	[5]
Laras *et al.*	Community-based	Febrile	Yes	2001	Bogor	Cross-sectional	Chikungunya suspect patients	86	58	67.4	IgG	Low	NA	Low	Unknown	Low	[5]
Laras *et al.*	Community-based	Non-febrile	Yes	2001	Bogor	Cross-sectional	Healthy individuals	70	35	50.0	IgG	Low	Low	Low	Unknown	Low	[5]
Laras *et al.*	Community-based	Non-febrile	No	2001	Bogor	Cross-sectional	Healthy individuals	99	13	13.1	IgG	Low	Low	Low	Unknown	Low	[5]
Laras *et al.*	Unknown	Febrile	Yes	2002-2003[b]	North Sulawesi	Unknown	Chikungunya suspect patients	222	18	8.1	IgG	Low	NA	Low	Unknown	High	[5]
Kanamitsu *et al.*	Unknown	Non-febrile	No	1972	Kalimantan	Cross-sectional	Healthy individuals	692	151	21.8	HI	Low	Unknown	Low	Unknown	High	[8]
Kanamitsu *et al.*	Unknown	Non-febrile	No	1972	Java	Cross-sectional	Healthy individuals	54	0	0.0	HI	Low	Unknown	Low	Unknown	Low	[8]
Kanamitsu *et al.*	Unknown	Non-febrile	No	1972	Bali	Cross-sectional	Healthy individuals	107	2	1.9	HI	Low	Unknown	Low	Unknown	High	[8]
Kanamitsu *et al.*	Unknown	Non-febrile	No	1972	Lombok	Cross-sectional	Healthy individuals	140	6	4.3	HI	Low	Unknown	Low	Unknown	High	[8]

Kanamitsu *et al.*	Unknown	Non-febrile	No	1972	Kupang	Cross-sectional	Healthy individuals	121	15	12.4	HI	Low	Unknown	Low	Unknown	High	[8]
Kanamitsu *et al.*	Unknown	Non-febrile	No	1972	Sulawesi	Cross-sectional	Healthy individuals	298	55	18.4	HI	Low	Unknown	Low	Unknown	High	[8]
Kanamitsu *et al.*	Unknown	Non-febrile	No	1972	Ambon	Cross-sectional	Healthy individuals	321	37	11.5	HI	Low	Unknown	Low	Unknown	High	[8]
Tesh *et al.*	Unknown	Non-febrile	No	1972	Kalimantan	Cross-sectional	Unknown	199	28	14.1	PRNT	Low	Unknown	Low	Unknown	High	[9]
Tesh *et al.*	Unknown	Non-febrile	No	1971	Sulawesi	Cross-sectional	Unknown	125	24	19.2	PRNT	Low	Unknown	Low	Unknown	High	[9]
Tesh *et al.*	Unknown	Non-febrile	No	1971	Ambon	Cross-sectional	Unknown	64	2	3.1	PRNT	Low	Unknown	Low	Unknown	Low	[9]
Tesh *et al.*	Unknown	Non-febrile	No	1969-1970	Papua	Cross-sectional	Unknown	243	45	18.5	PRNT	Low	Unknown	Low	Unknown	High	[9]

Table D. Individual characteristics of included studies on Chikungunya virus genotypes circulating in Indonesia

Study	Presentation	Outbreak	Year	Location	Design	Setting	Cases	Diagnosis	Sample	Origin	Number of sequences	Genotype(s)	Accession number (GenBank)	Risk of bias		Reference
														Isolate	Location & Date	
Sasmono *et al*.	Febrile	No	2015	Jambi	Cross-sectional	Local cases	DEN-like illness	alphavirus-specific PCR	103 tested	Human	8	Asian	KX097981-KX097988	Low	Low	[10]
Sari *et al*.	Febrile	Yes	2016	Bali	Cross-sectional	Local cases	Acute febrile	PCR or CHIKV-specific IgM	15 tested	Human	6	Asian	KY885022-KY885027	Low	Low	[3]
Yang *et al*.	Febrile	Unknown	2007-2014	Indonesia	Cross-sectional	Exported case	CF and DF suspected cases	RT-PCR, IgM and IgG, Isolation	Unknown	Human	29	ECSA (6), Asian (23)	KU561427-KU561432 and KU561436-KU561458	Low	Low	[11]
Riswari *et al*.	Febrile	No	2007	Bandung	retrospective cohort study	Local cases	Non-DENV acute febrile	RT-PCR, isolation, IgM	5	Human	3	Asian	KT175539-KT175541	Low	Low	[12]
Wolfel *et al*.	Febrile	Unknown	2012	Bali	Cross-sectional	Exported case	Chikungunya fever case	Unknown	1	Human	1	Asian	KM673291	Low	Low	[13]
Maha *et al*.	Febrile	Yes	2011	Mataram, Jembrana, Tangerang, Ngawi, Kubu Raya	Cross-sectional	Local cases	Chikungunya-suspect case	PCR (RT-PCR	109 tested	Human	28	ECSA (2), Asian (26)	KJ729829-KJ729856	Low	Low	[14]
Shchelkanov *et al*.	Febrile	Unknown	2013	Indonesia	Cross-sectional	Exported case	Chikungunya fever case	Unknown	1	Human	1	Asian	KF872195	Low	Low	[15]
Scholte *et al*.	Febrile	Unknown	2010	Indonesia	Cross-sectional	Exported case	Chikungunya-suspect case	Unknown	1	Human	1	ECSA	KC862329	Low	Low	[16]
Kosasih *et al*.	Febrile	No	2000-2008	Bandung	prospective	Local cases	Non-dengue febrile illness	IgM, IgG, RT-PCR, isolation	47 positive RT-PCR	Human	20	Asian	KC879559-KC879578	Low	Low	[1]
Mulyatno *et al*.	NA	No	2011	Surabaya	Cross-sectional	Local mosquitos	NA	Culture and RT-PCR	29 pools	Ae. aegypti male	2	Asian	AB678689-AB678690	Low	Low	[17]
Mulyatno *et al*.	Febrile	No	2010-2011	Surabaya	Cross-sectional	Local cases	DF or CHIKF diagnosed cases	Culture and RT-PCR	773 cultured	Human	17	Asian	AB678691-AB678695 AB678678-AB678688	Low	Low	[17]

Study	Presentation	Outbreak	Year	Location	Design	Setting	Cases	Diagnosis	Sample	Origin	Number of sequences	Genotype(s)	Accession number (GenBank)	Risk of bias		Reference
														Isolate	Location & Date	
Grandadam *et al*.	Unknown	Unknown	2009	Indonesia	Cross-sectional	Exported case	Unknown	Unknown	Unknown	Human	1	Asian	FR846307	Low	Low	[18]
Volk *et al*.	Unknown	Unknown	1983	Indonesia	Unknown	Unknown	Unknown	Unknown	Unknown	Human	1	Asian	HM045791	Low	Low	[19]
Volk *et al*.	Unknown	Unknown	1985	Indonesia	Unknown	Unknown	Unknown	Unknown	Unknown	Human	1	Asian	HM045797	Low	Low	[19]
Ng *et al*.	Febrile	Unknown	2008	Indonesia	Cross-sectional	Exported case	Suggestive of CHIKF cases	RT-PCR and IgM	Unknown	Human	2	ECSA (1), Asian (1)	FJ445483, FJ445472	Low	Low	[20]
Huang *et al*.	Febrile	Unknown	2007-2008	Indonesia	Cross-sectional	Exported case	Fever cases	RT-PCR	Unknown	Human	7	Asian	FJ807897, FJ807886-FJ807891	Low	Low	[21]
Shu *et al*.	Febrile	Unknown	2007	Indonesia	Cross-sectional	Exported case	Fever cases	RT-PCR, IgM, IgG, IgA	Unknown	Human	1	Asian	EU192143	Low	Low	[22]
Powers *et al*.	Unknown	Unknown	1985	Ambon	Unknown	Local cases	Unknown	Unknown	Unknown	Human	1	Asian	AF192894	Low	Low	[23]

Table E. Individual characteristics of included studies on exported chikungunya cases originating from Indonesia

Study	Presentation	Year(s)	Country of reporting	Design	Setting	Sample tested	Number of confirmed cases	Diagnosis method	Genotype (n)	Risk bias		Reference
										Travel history	Diagnosis method	
Knope *et al*.	Febrile	2013-2014	Australia	Cross-sectional	Passive surveillance system	Unknown	47	IgM, IgG, RT-PCR and isolation	NA	Low	Low	[24]
Yang *et al*.	Febrile	2012-2014	Taiwan	Cross-sectional	Active and passive surveillance system	Unknown	23	RT-PCR, isolation and sequencing	Asian (15) and unidentified (8)	Low	Low	[11]
Knope *et al*.	Febrile	2012-2013	Australia	Cross-sectional	Passive surveillance system	Unknown	34	IgM, IgG, RT-PCR and isolation	NA	Low	Low	[25]
Yang *et al*.	Febrile	2009-2010	Taiwan	Cross-sectional	Active and passive surveillance system	Unknown	16	RT-PCR, isolation and sequencing	Asian (8), ECSA (6) and unidentified (2)	Low	Low	[11]
Wolfel *et al*.	Febrile	2012	Germany	Cross-sectional	Case-study	1	1	Isolation and sequencing	Asian (1)	Low	Low	[13]
Knope *et al*.	Febrile	2011-2012	Australia	Cross-sectional	Passive surveillance system	Unknown	2	IgM, IgG, RT-PCR and isolation	NA	Low	Low	[26]
Shchelkanov *et al*.	Febrile	2013	Russia	Cross-sectional	Case-study	1	1	RT-PCR, isolation and sequencing	Asian (1)	Low	Low	[15]
Roth *et al*.	Unknown	2013	New Caledonia	Unknown	Unknown	Unknown	1	RT-PCR and sequencing	Asian (1)	Low	Low	[27]
Knope *et al*.	Febrile	2010-2011	Australia	Cross-sectional	Passive surveillance system	Unknown	32	IgM, IgG, RT-PCR and isolation	NA	Low	Low	[28]
Cha *et al*.	Febrile	2009-2010	South Korea	Cross-sectional	A part of dengue surveillance	Unknown	3	IgM	NA	Low	Low	[29]
Scholte *et al*.	Febrile	2010	Netherlands	Cross-sectional	Unknown	1	1	Isolation and sequencing	ECSA (1)	Low	Low	[16]
Dupont-Rouzeyrol *et al*.	Febrile	2011	New Caledonia	Unknown	Unknown	Unknown	2	Serology	NA	High	Low	[30]
Chaves *et al*.	Febrile	2010	Brazil	Cross-sectional	Case-study	1	1	IgM, IgG	NA	Low	Low	[31]
Albuquerque *et al*.	Febrile	2010	Brazil	Cross-sectional	Case-study	1	1	IgM and HI test	NA	Low	Low	[32]
Wright *et al*.	Febrile	2009-2010	Australia	Cross-sectional	Passive surveillance system	Unknown	7	IgM, IgG, RT-PCR and isolation	NA	Low	Low	[33]
Mizuno *et al*.	Febrile	2009	Japan	Cross-sectional	Case-study	3	3	IgM and PRNT	NA	Low	Low	[34]
Grandadam *et al*.	Febrile	2009	France	Cross-sectional	Unknown	1	1	IgM, RT-PCT and sequencing	Asian (1)	Low	Low	[18]

Study	Presentation	Year(s)	Country of reporting	Design	Setting	Sample tested	Number of confirmed cases	Diagnosis method	Genotype (n)	Risk bias		Reference
										Travel history	Diagnosis method	
Kobashi *et al*.	Febrile	2010	Japan	Cross-sectional	Case-study	1	1	IgM and RT-PCR	NA	Low	Low	[35]
Fitzsimmons *et al*.	Febrile	2008-2009	Australia	Cross-sectional	Passive surveillance system	Unknown	2	IgM, IgG, RT-PCR and isolation	NA	Low	Low	[36]
Huang *et al*.	Febrile	2006-2009	Taiwan	Cross-sectional	Active surveillance system	Unknown	7	RT-PCR, isolation and sequencing	Asian (7)	Low	Low	[21]
Ng *et al*.	Febrile	2009	Singapore	Cross-sectional	Active surveillance	Unknown	3	IgM, RT-PCR and sequencing	Asian (1), ECSA (1)	Low	Low	[20]
Fitzsimmons *et al*.	Febrile	2007	Australia	Cross-sectional	Passive surveillance system	Unknown	3	IgM, IgG and RT-PCR	NA	Low	Low	[37]
Shu *et al*.	Febrile	2007	Taiwan	Cross-sectional	Active surveillance system	Unknown	1	RT-PCR, isolation and sequencing	Asian (1)	Low	Low	[22]
Panning *et al*.	Febrile	2006	Europe	Cross-sectional	Passive surveillance system	Unknown	1	IgM, IgG or RT-PCR	NA	Low	Low	[38]
Harnett and Bucens	Febrile	1989	Australia	Cross-sectional	Case-study	1	1	IgM, IgG and isolation	NA	Low	Low	[39]

Reference

1. Kosasih H, de Mast Q, Widjaja S, Sudjana P, Antonjaya U, Ma'roef C, Riswari SF, Porter KR, Burgess TH, Alisjahbana B *et al*: Evidence for endemic chikungunya virus infections in Bandung, Indonesia. *PLoS neglected tropical diseases* 2013, 7(10):e2483.
2. Capeding MR, Chua MN, Hadinegoro SR, Hussain, II, Nallusamy R, Pitisuttithum P, Rusmil K, Thisyakorn U, Thomas SJ, Huu Tran N *et al*: Dengue and other common causes of acute febrile illness in Asia: an active surveillance study in children. *PLoS neglected tropical diseases* 2013, 7(7):e2331.
3. Sari K, Myint K, Andayani A, Adi P, Dhenni R, Perkasa D, Maroef C, Witaria N, Megawati D, Powers A *et al*: Chikungunya fever outbreak identified in North Bali, Indonesia. *Trans R Soc Trop Med Hyg* 2017, [In press].
4. Ngwe T, Inoue S, Thant KZ, Talemaitoga N, Aryati A, Dimaano EM, Matias RR, Buerano CC, Natividad FF, Abeyewickreme W *et al*: Retrospective seroepidemiological study of chikungunya infection in South Asia, Southeast Asia and the Pacific region. *Epidemiology and infection* 2016, 144(11):2268-2275.
5. Laras K, Sukri NC, Larasati RP, Bangs MJ, Kosim R, Djauzi, Wandra T, Master J, Kosasih H, Hartati S *et al*: Tracking the re-emergence of epidemic chikungunya virus in Indonesia. *Trans R Soc Trop Med Hyg* 2005, 99(2):128-141.
6. Porter KR, Tan R, Istary Y, Suharyono W, Sutaryo, Widjaja S, Ma'Roef C, Listiyaningsih E, Kosasih H, Hueston L *et al*: A serological study of Chikungunya virus transmission in Yogyakarta, Indonesia: evidence for the first outbreak since 1982. *The Southeast Asian journal of tropical medicine and public health* 2004, 35(2):408-415.
7. Suharti C, van Gorp EC, Dolmans WM, Groen J, Hadisaputro S, Djokomoeljanto RJ, D MEO, van der Meer JW: Hanta virus infection during dengue virus infection outbreak in Indonesia. *Acta medica Indonesiana* 2009, 41(2):75-80.
8. Kanamitsu M, Taniguchi K, Urasawa S, Ogata T, Wada Y, Saroso JS: Geographic distribution of arbovirus antibodies in indigenous human populations in the Indo-Australian archipelago. *The American journal of tropical medicine and hygiene* 1979, 28(2):351-363.
9. Tesh RB, Gajdusek DC, Garruto RM, Cross JH, Rosen L: The distribution and prevalence of group A arbovirus neutralizing antibodies among human populations in Southeast Asia and the Pacific islands. *The American journal of tropical medicine and hygiene* 1975, 24(4):664-675.
10. Sasmono R, Perkasa A, Yohan B, Haryanto S, Yudhaputri FA, Hayati R, Maaroef C, Ledermann J, Myint K, Powers A: Chikungunya deterection during dengue outbreak in Sumatera, Indonesia: Clinical manifestatation and virological profile. *The American journal of tropical medicine and hygiene* 2017, 97(5):1393-1398.
11. Yang CF, Su CL, Hsu TC, Chang SF, Lin CC, Huang JC, Shu PY: Imported chikungunya virus strains, Taiwan, 2006-2014. *Emerging infectious diseases* 2016, 22(11):1981-1984.
12. Riswari SF, Ma'roef CN, Djauhari H, Kosasih H, Perkasa A, Yudhaputri FA, Artika IM, Williams M, van der Ven A, Myint KS *et al*: Study of viremic profile in febrile specimens of chikungunya in Bandung, Indonesia. *Journal of clinical virology : the official publication of the Pan American Society for Clinical Virology* 2016, 74:61-65.
13. Wolfel S, Vollmar P, Poluda D, Zange S, Antwerpen MH, Loscher T, Dobler G: Complete genome sequence of a chikungunya virus imported from Bali to Germany. *Genome announcements* 2015, 3(2):e00164-00115.
14. Maha MS, Susilarini NK, Hariastuti NI: Chikungunya virus mutation, Indonesia, 2011. *Emerging infectious diseases* 2015, 21(2):379-381.
15. Shchelkanov M, L'Vov D K, Kolobukhina LV, Al'khovskii SV, Shchetinin AM, Saifullin MA, Kruzhkova IS, Aristova VA, Morozova TV, Samokhvalov EI *et al*: [Isolation of the Chikungunya virus in Moscow from the Indonesian visitor (September, 2013)]. *Voprosy virusologii* 2014, 59(3):28-34.
16. Scholte FE, Tas A, Martina BE, Cordioli P, Narayanan K, Makino S, Snijder EJ, van Hemert MJ: Characterization of synthetic Chikungunya viruses based on the consensus sequence of recent E1-226V isolates. *Plos One* 2013, 8(8):e71047.
17. Mulyatno KC, Susilowati H, Yamanaka A, Soegijanto S, Konishi E: Primary isolation and phylogenetic studies of Chikungunya virus from Surabaya, Indonesia. *Japanese journal of infectious diseases* 2012, 65(1):92-94.
18. Grandadam M, Caro V, Plumet S, Thiberge JM, Souares Y, Failloux AB, Tolou HJ, Budelot M, Cosserat D, Leparc-Goffart I *et al*: Chikungunya virus, southeastern France. *Emerging infectious diseases* 2011, 17(5):910-913.
19. Volk SM, Chen R, Tsetsarkin KA, Adams AP, Garcia TI, Sall AA, Nasar F, Schuh AJ, Holmes EC, Higgs S *et al*: Genome-scale phylogenetic analyses of chikungunya virus reveal independent emergences of recent epidemics and various evolutionary rates. *Journal of virology* 2010, 84(13):6497-6504.
20. Ng LC, Tan LK, Tan CH, Tan SS, Hapuarachchi HC, Pok KY, Lai YL, Lam-Phua SG, Bucht G, Lin RT *et al*: Entomologic and virologic investigation of chikungunya, Singapore. *Emerging infectious diseases* 2009, 15(8):1243-1249.
21. Huang JH, Yang CF, Su CL, Chang SF, Cheng CH, Yu SK, Lin CC, Shu PY: Imported chikungunya virus strains, Taiwan, 2006-2009. *Emerging infectious diseases* 2009, 15(11):1854-1856.
22. Shu PY, Yang CF, Su CL, Chen CY, Chang SF, Tsai KH, Cheng CH, Huang JH: Two imported chikungunya cases, Taiwan. *Emerging infectious diseases* 2008, 14(8):1326-1327.
23. Powers AM, Brault AC, Tesh RB, Weaver SC: Re-emergence of Chikungunya and O'nyong-nyong viruses: evidence for distinct geographical lineages and distant evolutionary relationships. *The Journal of general virology* 2000, 81(Pt 2):471-479.
24. Knope KE, Muller M, Kurucz N, Doggett SL, Feldman R, Johansen CA, Hobby M, Bennett S, Lynch S, Sly A *et al*: Arboviral diseases and malaria in Australia, 2013-14: Annual report of the National Arbovirus and Malaria Advisory Committee. *Commun Dis Intell Q Rep* 2016, 40(3):E400-E436.
25. Knope KE, Kurucz N, Doggett SL, Muller M, Johansen CA, Feldman R, Hobby M, Bennett S, Sly A, Lynch S *et al*: Arboviral diseases and malaria in Australia, 2012-13: Annual report of the National Arbovirus and Malaria Advisory Committee. *Commun Dis Intell Q Rep* 2016, 40(1):E17-47.

26. Knope KE, Doggett SL, Kurucz N, Johansen CA, Nicholson J, Feldman R, Sly A, Hobby M, El Saadi D, Muller M *et al*: Arboviral diseases and malaria in Australia, 2011-12: Annual report of the National Arbovirus and Malaria Advisory Committee. *Commun Dis Intell Q Rep* 2014, 38(2):E122-142.
27. Roth A, Hoy D, Horwood PF, Ropa B, Hancock T, Guillaumot L, Rickart K, Frison P, Pavlin B, Souares Y: Preparedness for threat of chikungunya in the pacific. *Emerging infectious diseases* 2014, 20(8).
28. Knope K, Whelan P, Smith D, Johansen C, Moran R, Doggett S, Sly A, Hobby M, Kurucz N, Wright P *et al*: Arboviral diseases and malaria in Australia, 2010-11: Annual report of the National Arbovirus and Malaria Advisory Committee. *Commun Dis Intell Q Rep* 2013, 37(1):E1-E20.
29. Cha GW, Cho JE, Lee EJ, Ju YR, Han MG, Park C, Jeong YE: Travel-associated chikungunya cases in South Korea during 2009-2010. *Osong public health and research perspectives* 2013, 4(3):170-175.
30. Dupont-Rouzeyrol M, Caro V, Guillaumot L, Vazeille M, D'Ortenzio E, Thiberge JM, Baroux N, Gourinat AC, Grandadam M, Failloux AB: Chikungunya virus and the mosquito vector Aedes aegypti in New Caledonia (South Pacific Region). *Vector Borne Zoonotic Dis* 2012, 12(12):1036-1041.
31. Chaves T, Pellini A, Mascheretti M, Jahnel M, Ribeiro A, Rodrigues S, Vasconcelos P, Boulos M: Travelers as sentinels for chikungunya fever, Brazil. *Emerging infectious diseases* 2012, 18(3):529-530.
32. Albuquerque IG, Marandino R, Mendonca AP, Nogueira RM, Vasconcelos PF, Guerra LR, Brandao BC, Aguiar GR, Bacco PA: Chikungunya virus infection: report of the first case diagnosed in Rio de Janeiro, Brazil. *Revista da Sociedade Brasileira de Medicina Tropical* 2012, 45(1):128-129.
33. Wright P, Fitzsimmons GJ, Johansen CA, Whelan PI, Co NAMA: Arboviral diseases and malaria in Australia, 2009-10: Annual report of the National Arbovirus and Malaria Advisory Committee. *Commun Dis Intell Q Rep* 2012, 36(1):70-81.
34. Mizuno Y, Kato Y, Takeshita N, Ujiie M, Kobayashi T, Kanagawa S, Kudo K, Lim CK, Takasaki T: Clinical and radiological features of imported chikungunya fever in Japan: A study of six cases at the National Center for Global Health and Medicine. *Journal of infection and chemotherapy : official journal of the Japan Society of Chemotherapy* 2011, 17(3):419-423.
35. Kobashi K, Kobayashi T, Nakamura-Uchiyama F, Ohnishi K: [A Japanese patient with chikungunya fever returning from Flores Island, Indonesia]. *Kansenshogaku Zasshi* 2010, 84(4):457-459.
36. Fitzsimmons GJ, Wright P, Johansen CA, Whelan PI, Arbovirus N: Arboviral diseases and malaria in Australia, 2008-09: Annual report of the National Arbovirus and Malaria Advisory Committee. *Commun Dis Intell Q Rep* 2010, 34(3):225-240.
37. Fitzsimmons GJ, Wright P, Johansen CA, Whelan PI, Ad NAM: Arboviral diseases and malaria in Australia, 2007/08: Annual report of the National Arbovirus and Malaria Advisory Committee. *Commun Dis Intell Q Rep* 2009, 33(2):155-169.
38. Panning M, Grywna K, van Esbroeck M, Emmerich P, Drosten C: Chikungunya fever in travelers returning to Europe from the Indian Ocean region, 2006. *Emerging infectious diseases* 2008, 14(3):416-422.
39. Harnett GB, Bucens MR: Isolation of chikungunya virus in Australia. *The Medical journal of Australia* 1990, 152(6):328-329.
